# A discrete model for analyzing the free vibrations of a non-uniform 2D-FGM beam under elastic foundations and different support conditions

**DOI:** 10.1038/s41598-025-32206-4

**Published:** 2025-12-23

**Authors:** Anass Moukhliss, Abdellatif Rahmouni, Rhali Benamar

**Affiliations:** 1 National Higher School of Electricity and Mechanics, ENSEM, Hassan II University of Casablanca, B.P 8118 Oasis Casablanca, Morocco; 2 Laboratory of Mechanics, Production and Industrial Engineering, LMPGI, Higher School of Technology of Casablanca, ESTC, Hassan II University of Casablanca, B.P 8112 Oasis Casablanca, Morocco; 3 Laboratoire des Etudes et Recherches en Simulation, Instrumentation et Mesures LERSIM, Mohammed V University of Rabat-Mohammadia School of Engineering, Avenue Ibn Sina, Morocco

**Keywords:** Engineering, Mathematics and computing, Physics

## Abstract

This paper proposes a discrete physical model (DPM) for the transverse free vibrations of non-uniform bi-directional functionally graded (2D-FGM) beams. The material properties vary in both the axial and thickness directions according to exponential laws, and the beam rest on a spatially variable elastic foundation and satisfy general support conditions. In the proposed formulation, the continuous beam is replaced by a multi-degree-of-freedom chain of lumped masses connected by bars and linear rotational and vertical springs. An adaptive discretization strategy is employed to construct consistent mass, stiffness, and foundation matrices. By applying Hamilton’s principle, the governing equations are reduced to an algebraic eigenvalue problem, from which nondimensional natural frequencies and associated mode shapes are obtained. Comparison with published results confirms the accuracy and reliability of the DPM. Owing to its simplicity and low computational cost, the model is well suited for extensive parametric studies and design-oriented analyses, including frequency tuning through geometric parameters, the material gradation exponents $(n_x, n_y)$, the taper ratio $\gamma$, and foundation characteristics (position $\chi$, intensity $\eta$) under various boundary conditions. The proposed approach provides a practical and efficient tool for analyzing and optimizing complex FGM beam structures.

## Introduction

In recent years, Functionally Graded Materials (FGMs) have garnered significant interest from the engineering and research communities. Due to their material properties that vary continuously in space at the macroscopic scale, FGMs exhibit remarkably high strength-to-weight and stiffness-to-weight ratios^1^. They have been successfully utilised in civil engineering, aerospace, and other technological applications, generally outperforming conventional fibre–matrix materials in terms of mechanical behaviour. In parallel, graded and composite media are increasingly used in advanced structural components and coatings, for example in corrugated composite plates where shear effects must be accurately captured^2^, or in functionally graded piezoelectric-piezomagnetic (FGPM) coating-substrate systems subjected to fretting contact under cyclic torque^3^.

The growing use of graded and multifunctional materials in dynamics and wave propagation further illustrates this trend. Recent works on SH-wave propagation in piezoelectric- piezomagnetic layered systems with reverse coupling and interfacial effects^4^, and on Love-type waves in ortho-viscoelastic and ortho-piezoelectric media with imperfect spring double-membrane interfaces^5^, highlight the need for accurate models that account for spatially varying properties and imperfect interfaces. At the structural scale, elastic wave modulation and vibroacoustic reduction have been achieved in beams with embedded oscillators and acoustic black-hole (ABH) type geometries^6,7^, while nonlinear vibration phenomena such as vortex-induced vibrations in bridge decks are now described using generalized multi-stable models^8^. In addition, several studies have emphasised the importance of soil-structure and foundation interaction in dynamic analysis, for instance in mitigating earthquake-induced pounding using viscoelastic devices^9^, in evaluating the vibration response of pile-raft foundations for ballastless tracks^10^, and in the discrete-element modelling of geosynthetic-reinforced pile-supported embankments^11^. Experimental and numerical investigations on the flexural behaviour of composite beams under cyclic loading^12^ further confirm that graded and composite members are often governed by complex vibration mechanisms that must be captured by reliable and computationally efficient models.

As the use of FGMs, whose material properties may vary along the length, the thickness, or both directions, continues to expand, there is a pressing need to develop reliable modelling strategies for their dynamic characterisation. In particular, efficient design and analysis techniques for structural components made from these materials are essential to enhance their performance against unwanted vibrations and to exploit the additional flexibility offered by graded properties, non-uniform geometries, and elastic foundations.

Khinchi et al.^13^ utilized the Higher-Order Differential Quadrature (HDQ) and Generalized Differential Quadrature (GDQ) methods to analyze the vibration characteristics of various configurations of FGM beams. Gantayat et al.^14^ employed the Finite Element Method (FEM) to predict the dynamic characteristics of graphene-reinforced Axially Functionally Graded (AFG) beams. Aubad et al.^15^ performed modal analysis and transient response assessments of AFG beams using FEM. Mahmoud^16^ applied the Myklestad method for analyzing free vibrations of tapered and stepped AFG beams with any number of attached masses. Bambaeechee et al.^17^ used analytical methods and design charts for the free vibration analysis of exponential AFGM beams with general boundary conditions and tip masses. Cao et al.^18^ proposed a novel approach for free vibration analysis of AFG beams with non-uniform cross-sections based on Chebyshev polynomial theory. Soltani et al.^19^ introduced a new hybrid approach for free vibration and stability analyses of AFG beams with variable cross-sections resting on a uniform Winkler-Pasternak foundation. Chen et al.^20^ explored the free transverse vibrational analysis of AFG tapered beams using the variational iteration approach. Nikolic and Aleksandar^21^ investigated the free vibration analysis of a non-uniform AFG cantilever beam with a tip mass. Mohammadnejad and Mehrdad^22^ analyzed the free vibration of axially functionally graded beams using Fredholm integral equations. Wang et al.^23^ employed a discrete model known as the Hencky Bar-Chain (HBC) model for buckling and vibration analyses of beams with elastic boundary conditions. Zhang et al.^24^ applied the same HBC model for buckling and vibration analyses of non-uniform beams on variable elastic foundations. Motaghian et al.^25^ developed a new Fourier series solution for the free vibration of non-uniform beams resting on variable elastic foundations. Particularly for beams composed of Bi-directional Functionally Graded Materials (2D-FGM). Tang et al.^26^ addressed nonlinear vibrations of Bi-directional Functionally Graded (2D-FGM) beams using the Generalized Differential Quadrature Method (GDQM) to determine vibration modes and nonlinear frequencies. Csimcsek and Mesut^27^ studied the free and forced vibrations of Timoshenko (2D-FGM) beams under various boundary conditions. Gartia et al.^28^ examined the free vibration of (2D-FGM) nanobeams resting on Winkler-Pasternak foundations, utilizing nonlocal strain gradient theory. Chen et al.^29^ conducted isogeometric analysis of (2D-FGM) porous micro-beams with geometrical imperfections using nonlocal strain gradient theory. Attia et al.^30^ investigated the vibrations of double-cracked bi-directional functionally graded nanobeams using the differential quadrature method. Li et al.^31^ employed meshless analysis of (2D-FGM) beams based on the physical neutral surface. Taima et al.^32^ performed a comparative study on the free vibration analysis of rotating bi-directional functionally graded beams using multiple beam theories, including Bernoulli-Euler (BE), Timoshenko (T), and Reddy (R) theories. Sharma et al.^33^ conducted a comparative study of the behavior of bi-directional functionally graded beams using two numerical techniques: the Harmonic Differential Quadrature (HDQ) and the Generalized Differential Quadrature (GDQ) methods to calculate the natural frequencies of the (2D-FGM) beams. Kumar et al.^34^ analyzed the dynamic behavior of bi-directional functionally graded beams with geometric nonlinearity using the domain method and FEM.

The study of non-uniform beams has emerged as a crucial area of research in structural mechanics, largely due to their wide-ranging applications in engineering. Non-uniform beams, characterised by variations in their cross-sectional dimensions along their length, offer significant advantages such as optimised weight distribution and improved structural performance under dynamic conditions^35^. These beams are integral to numerous structural elements, including bridge girders and turbine blades, where adaptability to varying loads is essential. However, despite the importance of these structures, the study of their dynamic behaviour, particularly when made of functionally graded materials (FGM) with bidirectional property gradients, remains relatively limited.

Extensive research has been conducted on the vibration characteristics of non-uniform beams. For example, Cao et al.^36^ applied an asymptotic perturbation approach to investigate the free vibration of non-uniform and non-homogeneous beams. Burlayenko et al.^37^ analysed porous beams with non-uniform rectangular cross-sections and explored four distinct porosity distributions across the thickness. While these studies are pivotal, they are limited to single-directional FGMs and often overlook the added complexity introduced by variable cross-sections. Similarly, Pekel et al.^38^ employed finite element analysis to determine the natural frequencies of non-uniform beams coated with FGM, while Ebrahimi et al.^39^ explored thermo-mechanical vibration behaviour of non-uniform beams under thermal loading. Further, Heshmati et al.^40^ extended this analysis to FGM porous Timoshenko beams, considering shear deformation and non-uniformity in the cross-section. Ghazaryan et al.^41^ used the differential transform method to study Euler-Bernoulli beams with non-uniform profiles and axially graded material properties under various boundary conditions. These studies highlight the complexity of non-uniform beams but do not focus on bidirectional FGMs.

In addition, Keshmiri et al.^42^ developed a method using the Adomian decomposition method to analyse the vibration mode shapes of tapered FGM beams. Hung and Young^43^ investigated the free vibration of non-uniform Timoshenko-Ehrenfest beams with arbitrary two-dimensional (2D-FGM) properties, demonstrating the complexity of such systems. Shanab et al.^44^ used the differential quadrature method to model the static bending, buckling, and vibration behaviours of tapered 2D-FGM micro/nanobeams, and Reddy et al.^45^ examined the vibration of 2D-FGM taper beams using a higher-order shear deformation theory. Despite these advances, the interaction between bidirectional material property variation, geometric variability, and complex boundary conditions in non-uniform beams remains underexplored.

While the above research highlights the progress made in the analysis of non-uniform beams, most existing studies focus on beams with unidirectional property variations. Very few address the complex interaction between bidirectional gradients and variable cross-sections. Furthermore, most research does not consider beams exposed to partially elastic and variable foundations, which are often encountered in real-world engineering applications. This gap is particularly evident when considering beams with complex profiles and different boundary conditions.

The novelty of the present research lies in its focus on non-uniform beams made of 2D-FGM materials, where both the mechanical properties and geometric dimensions vary. For the first time, this study considers beams exposed to variable elastic foundations, allowing for a more comprehensive understanding of their dynamic response.

In this study, a physical model is proposed to analyse the vibration of beams with varying cross-sectional profiles, characterised by a taper ratio $\gamma$. These beams are made of materials with bidirectional gradient properties (2D-FGM). The analysis employs a straightforward theory that yields information on natural non-dimensional frequencies and mode shapes. The beam is represented as a system comprising masses of magnitude $m_r$ for $r$ ranging from 1 to $N$, rotary springs with stiffness $C_r$ for $r = 1$ to $N + 2$, and bars spaced at equal intervals. Additionally, $N$ vertical springs are introduced to simulate the Winkler foundation, with stiffness $k^f_r$ for $r = 1$ to $N$. The physical properties of the beams are assumed to vary according to an exponential law distribution (E-FGM) of the volume fraction of their constituents.

Numerical results derived from this model are compared with other solutions available in the literature. The proposed theory is found to be both accurate and efficient in studying the dynamic response of beams with complex profiles and property gradients. Despite the linear nature of the model, addressing problems of this type remains a challenge, particularly when the beam is subjected to various types of foundations and boundary conditions. A significant advantage of this model is its simplicity and flexibility, enabling easier parametric studies that can be adapted to different configurations.

The research presented here offers a new perspective on the dynamic behaviour of beams made of bidirectionally graded materials, especially under conditions of variable elastic foundations. By simplifying the calculation process through a discrete model, this study contributes valuable insights into a domain that has remained underexplored, filling a critical gap in the understanding of non-uniform, 2D-FGM beams.

## Mechanical properties of the (2D-FGM) non-uniform beam

**Fig. 1:**
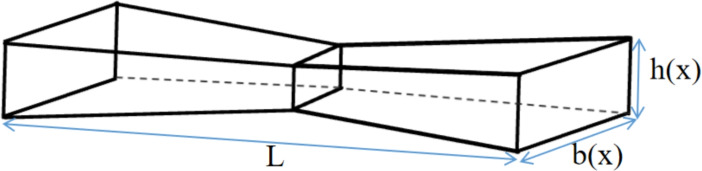
The non-uniform (2D-FGM )beam.

Fig 1 illustrates a non-uniform 2D Functionally Graded Material (2D-FGM) beam with a length *L*. The height and width of the beam are assumed to vary along its length, denoted by *h*(*x*) and *b*(*x*), respectively. The cross-sectional area and moment of inertia are represented by *S*(*x*) and *I*(*x*), respectively. The mechanical properties are considered to vary continuously in both the longitudinal and height directions of the (2D-FGM) beam following an exponential variation law :

1 $$\begin{aligned} & E(x,y) = \left( {\left( {{E_c} - {E_m}} \right) \frac{{\left( {\exp \left( {n_x}\frac{x}{L} \right) - 1} \right) }}{{\left( {\exp ({n_x}) - 1} \right) }} + {E_m}} \right) \exp \left( {{n_y}\frac{y}{h(x)}} \right) \end{aligned}$$

2 $$\begin{aligned} & \rho (x,y) = \left( {\left( {{\rho _c} - {\rho _m}} \right) \frac{{\left( {\exp \left( {n_x}\frac{x}{L} \right) - 1} \right) }}{{\left( {\exp ({n_x}) - 1} \right) }} + {\rho _m}} \right) \exp \left( {{n_y}\frac{y}{h(x)}} \right) \end{aligned}$$

In this context, $E_m$, $\rho _m$, $E_c$, and $\rho _c$ represent the material properties of the beam, which is composed of aluminum and ceramic. The properties for aluminum are $E_m = 70 \, \text {GPa}$ and $\rho _m = 2702 \, \text {kg/m}^3$, while for ceramic, they are $E_c = 200 \, \text {GPa}$ and $\rho _c = 5700 \, \text {kg/m}^3$. The parameters $n_x$ and $n_y$ denote the exponential variation of the material properties along the *x* and *y* axes, respectively. The height of the conical beam is defined as: $h(x) = h_{\text {max}} \left[ 1 - \gamma \left( 1 - \left| 1 - {2x}/{L} \right| \right) ^2 \right]$, where $h_{\text {max}}$ is the maximum height, $\gamma$ is the taper ratio, and *L* is the length of the beam.

**Fig. 2:**
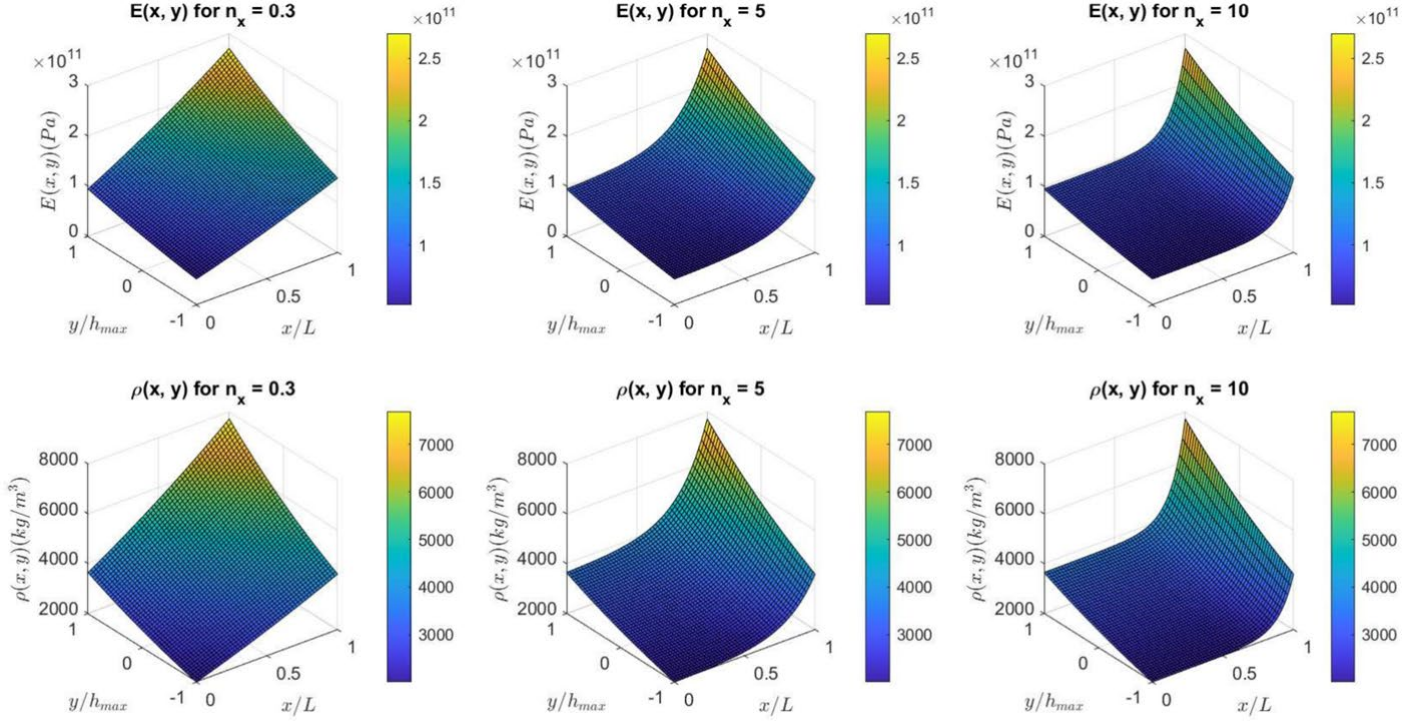
Variation of Young’s modulus and density for a uniform (2D-FGM) beam ($\gamma = 0$).

**Fig. 3:**
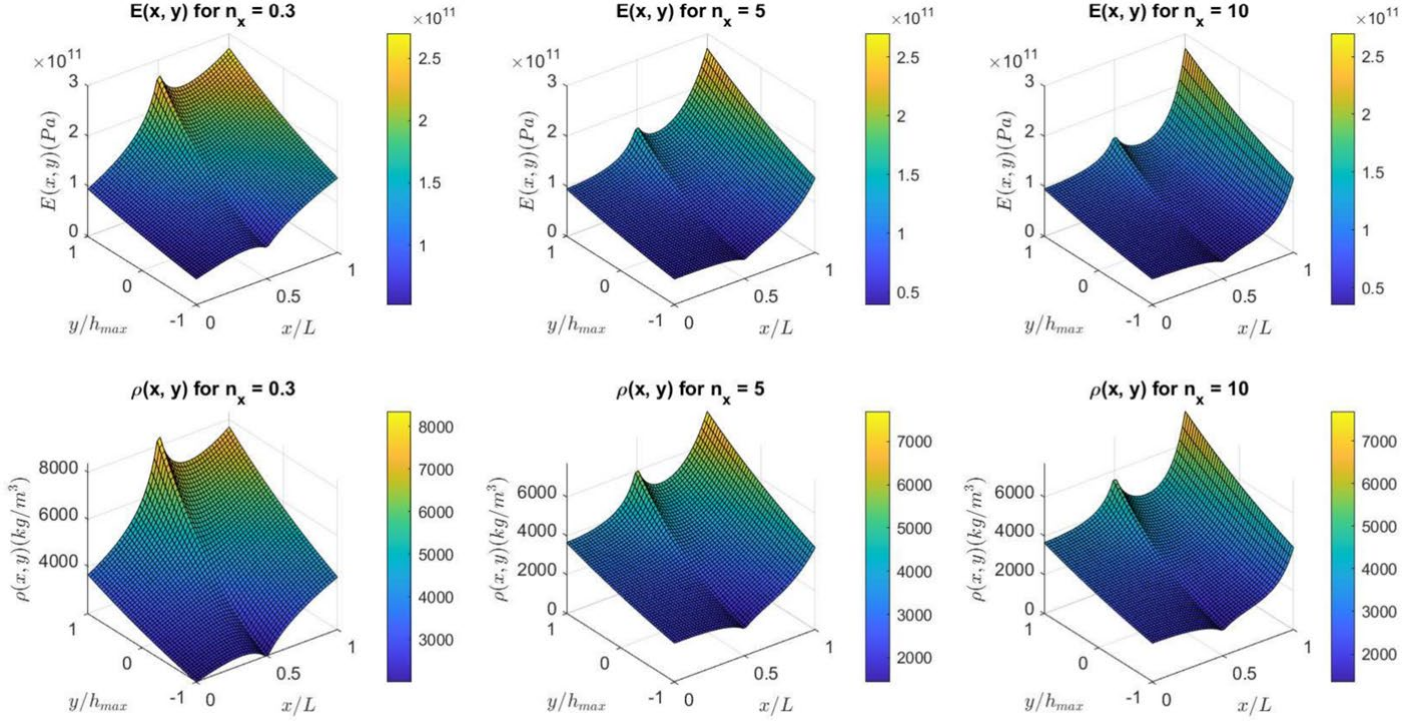
Variation of Young’s modulus and density for a non-uniform (2D-FGM) beam ($\gamma = 0.8$).

Figures 2 and 3 illustrate the variation in Young’s modulus and density of a non-uniform (2D-FGM) beam for $\gamma = 0$ and $\gamma = 0.6$, respectively. The gradation index chosen here is $n_y = 0.3$, with different gradation indices of $n_x = 0.3$, $n_x = 5$, and $n_x = 10$.

## Discrete model approach

### Modelling techniques

**Fig. 4:**
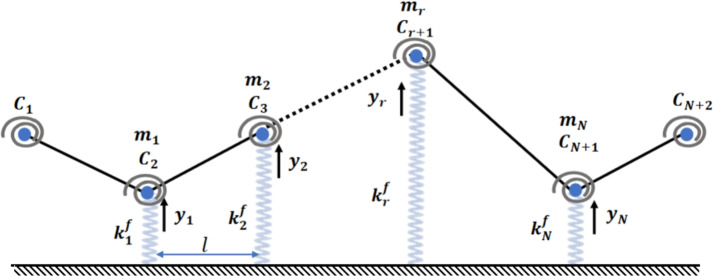
Discrete physical model containing masses, bars, and spiral springs.

This discrete model describes the beam as a system with (MDOF), composed of *N* masses $m_r$, $(N+1)$ small undeformable bars of length *l*, mass and zero inertia. The system also includes $(N+2)$ spiral springs with coefficients $C_r$ simulating the rigidity due to the beam’s flexure, as depicted in Fig 4. It is important to note that part of the beam’s mass near the fixed ends does not contribute to kinetic energy, ensuring that the distance *l* between two masses is $L/(N+1)$.

In this model, shear effects are negligible, assuming that the beam follows the Euler-Bernoulli theory. Under this hypothesis, the bending moment can be expressed as $M_r = C_r \Delta \theta _r$, where $\Delta \theta _r = \theta _{r} - \theta _{r-1}$ represents the angular variation near node *r* for $r=1,...,N+2$. The stiffness coefficients $C_1$ and $C_{N+2}$ are adjusted to model the flexibility of the beam ends under various boundary conditions. For a simply supported (SS) beam $(C_1 = C_{N+2} = 0)$, for a clamped-clamped (CC) beam $(C_1 = C_{N+2} = \infty )$, for a clamped-simply supported (CS) beam $(C_1 = \infty ,\, C_{N+2} = 0)$, and for a clamped-free (CF) beam $(C_1 = \infty ,\, C_{N+1} = C_{N+2} = 0)$^46,47^.

The beam considered rests on a partially elastic Winkler foundation. Two types of foundations are studied with distinct laws :

3 $$\begin{aligned} & {k^{1f}}(x) = \frac{{{E_m}{I_0}}}{{{L^4}}}\eta {(1 - \alpha x/L)^2} \end{aligned}$$

4 $$\begin{aligned} & {k^{2f}}(x) = \frac{{{E_m}{I_0}}}{{{L^4}}}\eta (1 + \sin (\alpha x/L)) \end{aligned}$$

$\eta$ and $\alpha$ represent the dimensionless stiffness of the foundation and the periodicity, respectively, and $I_0$ is the moment of inertia at $x = 0$.

The elastic foundation of the soil column is modelled in the (DPM) by *N* vertical springs with stiffness $k_r^f = k^{sf}(x_r) l$, for $r=1, \ldots , N$^48^, as shown in Fig  4. These stiffness values are determined based on the law describing the variation of the foundation stiffness $k^{sf}(x)$ with $s = 1$ or 2.

### Equations of motion

For the (DPM) shown in Fig 4, the potential energy related to bending, the potential energy of the foundation, and the kinetic energy are described in tensor notation by Eqs. 5, 6, and 7, respectively^46,48–52^.

5 $$\begin{aligned} & V_l = \frac{k^l_{ij}}{2} y_i y_j \end{aligned}$$

6 $$\begin{aligned} & V_f = \frac{k^f_{ij}}{2} y_i y_j \end{aligned}$$

7 $$\begin{aligned} & T = \frac{m_{ij}}{2} \dot{y_i} \dot{y_j} \end{aligned}$$

The components $m_{ij}$ denote the mass tensor. The stiffness tensor associated with the elastic foundation, represented by $k^f_{ij}$ and $k^l_{ij}$, also represents the system’s linear stiffness tensor.

Using the Hamiltonian approach:

8 $$\begin{aligned} \delta \int (V_l + V_f - T) \, dt = 0 \end{aligned}$$

This leads to the equations of motion for the (DPM):

9 $$\begin{aligned} m_{ir} \ddot{y}_i + k^l_{ir} y_i + k^f_{ir} y_i = 0 \quad \text {for } r = 1, \ldots , N \end{aligned}$$

Equation 9 can be represented in matrix form as:

10 $$\begin{aligned} [M] \{\ddot{y}\} + ([K^f]+[K^l]) \{y\} = 0 \end{aligned}$$

Here, [*M*], $[K^l]$, and $[K^f]$ represent the mass matrix, linear stiffness matrix of the (DPM), and foundation stiffness matrix, respectively.

The free response of the beam is assumed to be harmonic^46^ according to the following expression :

11 $$\begin{aligned} y_r = A_r \cos (\omega _{sys} t) \,\,\ \textrm{for} \,\,\,\ r=1,...,N \end{aligned}$$

Where $A_r$ is the amplitude of displacement $y_r$ of mass $m_r$ expressed in terms of displacement basis, and $\omega _{sys}$ is the vibration frequency corresponding to the (DPM).

Substituting Eq. 11 into Eq. 12, we get :

12 $$\begin{aligned} [K] \{A\} - \omega _{sys}^2 [M] \{A\} = 0 \end{aligned}$$

With $$[K]=[K^f]+[K^l]$$.

Next, the coefficients $m_{ij}$, $k^l_{ij}$, and $k^f_{ij}$ are calculated to solve the equation formulated in relation 12.

### Calculation of $m_{ij}$ , $k^l_{ij}$ and $k^f_{ij}$

We need to compute the revised mass tensor $m_{ij}$, as well as the coefficients of the linear stiffness tensor $k^l_{ij}$ and the foundation stiffness tensor $k^f_{ij}$, to accurately model the dynamic behaviour of the system.

#### The mass tensor $m_{ij}$

The general term of the mass tensor is defined as follows^53^:

13 $$\begin{aligned} {m_{ij}} = {m_i}{\delta _{ij}},\quad i,j = 1,...,N \end{aligned}$$

Where $\delta _{ij}$ is the Kronecker delta symbol.

The kinetic energy representing the vertical motion of a (2D-FGM) non- uniform beam is defined by the following expression:

14 $$\begin{aligned} T = \frac{1}{2}\int _0^L {\int _{ - h(x)/2}^{h(x)/2} {\int _{ - b(x)/2}^{b(x)/2} \rho } } (x,z){\left( {\frac{{\partial W(x,t)}}{{\partial t}}} \right) ^2}dS{\hspace{1.0pt}} dx \end{aligned}$$

This is also expressed as:

15 $$\begin{aligned} T = \frac{1}{2}\int _0^L {{I_0}} (x){\left( {\frac{{\partial W(x,t)}}{{\partial t}}} \right) ^2}dx \end{aligned}$$

In this context, ${I_0}(x) = \int \rho (x,y) \, dy \, dz$, with $\rho (x,y)$ indicating the mass density at position (*x*, *y*).

The relationship for calculating the mass density is given by:

16 $$\begin{aligned} {I_0}(x) = \frac{{2h(x)b(x)\sinh \left( {\frac{{n_y}}{2}} \right) }}{{{n_y}}}\left[ {\left( {{r_c} - {r_m}} \right) \frac{{\left( {\exp \left( {{n_x}\frac{x}{L}} \right) - 1} \right) }}{{\left( {\exp ({n_x}) - 1} \right) }} + {r_m}} \right] \end{aligned}$$

The elementary kinetic energy *dT* of a continuous beam element can be expressed as:

17 $$\begin{aligned} dT = \frac{1}{2}{I_0}(x){\left( {\frac{{\partial W}}{{\partial t}}} \right) ^2}dx \end{aligned}$$

A discrete modelling approach involves dividing the continuous beam into $(N+1)$ elements, considering that each element of length *dx* is replaced by a mass $m_r$ placed at node *r* in the discrete system, with the coresponding kinetic energy denoted as $T_r$ for $r = 1, \ldots , N$:

18 $$\begin{aligned} T_r = \frac{1}{2}I_0(x_r) \dot{y_r}^2 l \,\,\, \textrm{for}\,\,\, r=1,...,N \end{aligned}$$

With $I_0(x_r) = I_{0r}$, where $x_r$ represents the position of node *r* defined as $x_r = r \times l$. As previously mentioned, it is important to note that a portion of the beam’s mass near the fixed ends does not contribute to the kinetic energy. This ensures that the distance *l* between two masses is $L/(N+1)$.

The magnitude of the mass $m_r$ is defined as $m_r = I_{0r}$ for $r = 1, \ldots , N$. This relationship indicates that the continuous beam is discretized into point masses, whose magnitudes are dependent on the geometric characteristics of the continuous system.

The mass tensor corresponding to the (DPM) for modeling the (2D-FGM) non-uniform beam is given by:

19 $$\begin{aligned} {m_{ij}} =\frac{{L{\rho _m}{S_0}}}{{(N + 1)}} {m^*_i} {\delta _{ij}}\quad \mathrm{{for}}\quad i,j = 1, \ldots ,{N} \end{aligned}$$

In matrix form:

20 $$\begin{aligned} & \left[ M \right] = \frac{{L{\rho _m}{S_0}}}{{(N + 1)}}\left[ {\begin{array}{*{20}{c}} {m_1^*}& 0& 0& 0& 0& \ldots & .& \ldots & 0& 0\\ 0& \ddots & 0& 0& 0& \ddots & .& .& .& .\\ 0& 0& \ddots & 0& 0& 0& 0& .& \vdots & \vdots \\ 0& 0& 0& \ddots & \ddots & \ddots & 0& 0& \vdots & \vdots \\ 0& 0& 0& \ddots & \ddots & \ddots & \ddots & \ddots & 0& \vdots \\ .& .& 0& .& .& {m_r^*}& \ddots & \ddots & 0& 0\\ \vdots & .& 0& \ddots & .& \ddots & \ddots & \ddots & \ddots & 0\\ .& \vdots & .& \ddots & 0& \ddots & \ddots & {m_{N - 2}^*}& \ddots & 0\\ .& .& .& .& .& 0& \ddots & \ddots & \ddots & 0\\ 0& 0& \ldots & .& .& 0& 0& 0& 0& {m_N^*} \end{array}} \right] \end{aligned}$$

21 $$\begin{aligned} & \left[ M \right] = \frac{{L{\rho _m}{S_0}}}{{(N + 1)}}[{M^*}] \end{aligned}$$

Here, $[M^*]$ is the dimensionless mass matrix, which provides information on the distribution of the non-uniform beam’s mass among the *N* masses (accounting for the portion of the beam’s mass that does not contribute to kinetic energy).

For a non-uniform (2D-FGM) beam, the terms $m_r^*$ are defined as $m_r^* = I^*_{0r}$, where $x^*_r = {r}/{(N+1)}$ for $r = 1, \ldots , N$. The term $I^*_{0r}$ is given by:

22 $$\begin{aligned} I^*_{0r} = \frac{2h_r^* b_r^* \sinh \left( \frac{n_y}{2} \right) }{n_y} \left[ \left( \frac{r_c}{r_m} - 1 \right) \frac{\exp \left( n_x x_r^* \right) - 1}{\exp (n_x) - 1} + 1 \right] \end{aligned}$$

For a uniform and homogeneous beam, the terms $m_r^*$ for $r=1, \ldots , N$ are all equal to 1. In this case, the matrix $[M^*]$ becomes an identity matrix, denoted as $[M^*] = [I]$.

#### The linear stiffness tensor $k^l_{ij}$

This section details the expressions for the stiffness tensors in terms of the coefficients $C_r$ and derives the expression for $C_r$. Usually, the relationships involving angles $\theta _r$ are given as the sum of the derivatives of transverse displacements and shear rotation. Since curvature effects are considered minimal in this study, we adopt the method from^46^:

23 $$\begin{aligned} \sin \theta _r = \frac{(N+1)}{L}(y_r - y_{r-1}) \approx \theta _r, \quad r= 1,...,N + 1 \end{aligned}$$

The potential energy $V_l$ of the (DPM), which is due to the torsional forces acting in the $(N+2)$ linear spiral springs, can be determined by the following formula^46^:

24 $$\begin{aligned} {V_l} = \frac{{{{(N + 1)}^2}}}{{2{L^2}}}\sum \limits _{r = 1}^{N + 2} {{C_r}} {({y_{r - 2}} + {y_r} - 2{y_{r - 1}})^2} \end{aligned}$$

Where $$y_{-1} = y_0 = y_{N+1} = y_{N+2} = 0$$.

The mass $m_r$ is subjected to the effects of three nearby spiral springs located at nodes $(r)$, $(r+1)$, and $(r+2)$. The elastic mechanical force acting at node $r$ is expressed by^46^:

25 $$\begin{aligned} F_r^l = - \frac{1}{l^2} \left[ (y_r - 2y_{r-1} + y_{r-2}) C_r - 2 (y_{r+1} - 2y_r + y_{r-1}) C_{r+1} + (y_{r+2} - 2y_{r+1} + y_r) C_{r+2} \right] \end{aligned}$$

The force from the linear springs applied to mass $m_r$ can be derived from the linear potential energy $V_l$, as the force derives from potential energy, by taking a partial derivative with respect to coordinate $y_r$:

26 $$\begin{aligned} F_r^l = - \frac{\partial V_l}{\partial y_r} = - \frac{1}{2} \left[ y_j k_{rj} + y_i k_{ir} \right] = - y_i k_{ir} \quad \text {for} \quad i,j,r = 1,...,N \end{aligned}$$

In this expression, the classical symmetry relation, i.e., $k_{ij} = k_{ji}$, is adopted. Equations 25 and 26 lead to the expressions of the terms of the linear stiffness matrix :

27 $$\begin{aligned} & k_{(r-2)r} = \frac{C_r}{l^2} \quad \text {for} \quad r = 3,...,N \end{aligned}$$

28 $$\begin{aligned} & k_{(r-1)r} = - \frac{2}{l^2} (C_r + C_{r+1}) \quad \text {for} \quad r = 2,...,N \end{aligned}$$

29 $$\begin{aligned} & k_{rr} = \frac{1}{l^2} (C_r + 4C_{r+1} + C_{r+2}) \quad \text {for} \quad r = 1,...,N \end{aligned}$$

Other values are obtained through symmetry relations derived from Eqs. 27 to 29, while the rest are zero.

To determine the stiffness $C_r$ of the torsional springs, which represent the bending stiffness of the equivalent continuous (2D-FGM ) beam, we use the relation that links the flexural moment $M_{fr}$ in the spring to the rotation $\theta _r$^46,51^:

30 $$\begin{aligned} {M_{fr}} = {C_r}\Delta {\theta _r}\quad \mathrm{{with}}\quad r = 1, \ldots ,N + 2 \end{aligned}$$

On the other hand, the bending moment of a continuous (2D-FGM) Euler-Bernoulli beam is given by^26^:

31 $$\begin{aligned} {M_{f}} = \left( {{D_{11}}({x}) - \frac{{B_{11}^2({x})}}{{{A_{11}}({x})}}} \right) {\left( {\frac{{{\partial ^2}y}}{{\partial {x^2}}}} \right) } \end{aligned}$$

The following relationships are used to obtain the values of $A_{11}(x)$, $B_{11}(x)$, and $D_{11}(x)$ in Eq. 31, which represent the stretching stiffness coefficient, bending stiffness coefficient, and bending-stretching coupling stiffness coefficient, respectively^27^:

32 $$\begin{aligned} ({A_{11}}(x),{B_{11}}(x),{D_{11}}(x)) = \int _{ - h(x)/2}^{h(x)/2} {\int _{ - b(x)/2}^{b(x)/2} {{{E(x,y)}}} } (1,y,{y^2}){\hspace{1.0pt}} dy{\hspace{1.0pt}} dz \end{aligned}$$

The integral outcomes for the stiffness components of the materials in the non-uniform (2D-FGM) beam can be expressed as functions of the power law indices $n_x$ and $n_y$ as follows:

33 $$\begin{aligned} & {A_{11}}(x) = \frac{{2h(x)b(x)\sinh \left( {\frac{{n_y}}{2}} \right) }}{{{n_y}}}\left[ {\left( {{E_c} - {E_m}} \right) \frac{{\left( {\exp \left( {{n_x}\frac{x}{L}} \right) - 1} \right) }}{{\left( {\exp ({n_x}) - 1} \right) }} + {E_m}} \right] \end{aligned}$$

34 $$\begin{aligned} & {B_{11}}(x) = \frac{b(x) h(x)^2}{2 n_y^2} \exp \left( -\frac{n_y}{2} \right) \left( n_y - 2 \exp (n_y) + n_y \exp (n_y) + 2 \right) \nonumber \\ & \quad \times \left[ \left( E_c - E_m \right) \frac{\exp \left( n_x \frac{x}{L} \right) - 1}{\exp (n_x) - 1} + E_m \right] \end{aligned}$$

35 $$\begin{aligned} & {D_{11}}(x) = -\frac{b(x) h(x)^3}{4 n_y^3} \exp \left( -\frac{n_y}{2} \right) \left( 4 n_y - 8 \exp (n_y) - n_y^2 \exp (n_y) + 4 n_y \exp (n_y) + n_y^2 + 8 \right) \nonumber \\ & \quad \times \left[ \left( E_c - E_m \right) \frac{\exp \left( n_x \frac{x}{L} \right) - 1}{\exp (n_x) - 1} + E_m \right] \end{aligned}$$

Using the finite difference technique, we can write:

36 $$\begin{aligned} {\left( {\frac{{{d^2}y}}{{d{x^2}}}} \right) _r} = \frac{{y_r^\prime - y_{ r- 1}^\prime }}{l} = \frac{{{\theta _r} - {\theta _{r - 1}}}}{l} \end{aligned}$$

Substituting Eq. 36 into Eq. 31, we obtain the expression for the moment at node *r*:

37 $$\begin{aligned} {M_{fr}} = \left( {{D_{11}}({x_r}) - \frac{{B_{11}^2({x_r})}}{{{A_{11}}({x_r})}}} \right) \left( {\frac{{{\theta _r} - {\theta _{r - 1}}}}{l}} \right) \quad \mathrm{{for}}\quad r = 1,...,{N} + 2 \end{aligned}$$

The following adaptations to the notation are proposed: $A_{11}(x_r) = A_{11r}$, $B_{11}(x_r) = B_{11r}$, and $D_{11}(x_r) = D_{11r}$, consequently :

38 $$\begin{aligned} {M_{fr}} = \left( {{D_{11r}} - \frac{{B_{11r}^2}}{{{A_{11r}}}}} \right) \left( {\frac{{\Delta {\theta _r}}}{l}} \right) \quad \mathrm{{for}}\quad r = 1,...,{N}+2 \end{aligned}$$

From Eqs. 30 and 38, we deduce:

39 $$\begin{aligned} {C_r} = \frac{1}{l}\left( {{D_{11}}({x_r}) - \frac{{B_{11}^2({x_r})}}{{{A_{11}}({x_r})}}} \right) \quad \mathrm{{with}}\quad r = 2, \ldots ,{N} + 1 \end{aligned}$$

The stiffness values for $C_1$ and $C_{N+2}$ are determined based on the system’s boundary conditions.

To express the linear stiffness tensor in dimensionless terms, $C_r$ must be converted into its dimensionless counterpart. The dimensionless form of $C_r$, labeled $C^*_r$, is given by:

40 $$\begin{aligned} {C_r} = \frac{{{E_m}{I_0}}}{l}C_r^*\quad \mathrm{{with : }}\,\,C_r^* = D_{11r}^* - \frac{{{{\left( {B_{11r}^*} \right) }^2}}}{{A_{11r}^*}}\quad \mathrm{{for}}\quad r = 2, \ldots ,{N} + 1 \end{aligned}$$

Where $D_{11r}^*$, $B_{11r}^*$, and $A_{11r}^*$ are the dimensionless counterparts of $D_{11r}$, $B_{11r}$, and $A_{11r}$, respectively, and are expressed as follows:

41 $$\begin{aligned} & A_{11r}^* = \frac{{2h_r^*b_r^*\sinh ({n_y}/2)}}{{12{n_y}}}\left[ {\left( {\frac{{{E_c}/{E_m} - 1}}{{\exp ({n_x}) - 1}}} \right) \left( {\exp ({n_x}x_r^*) - 1} \right) + 1} \right] \end{aligned}$$

42 $$\begin{aligned} & B_{11r}^* = \frac{{b_r^*{{\left( {h_r^*} \right) }^2}\exp ( - {n_y}/2)}}{{2n_y^2}}\left( {{n_y} - 2\exp ({n_y}) + {n_y}\exp ({n_y}) + 2} \right) \left[ {\left( {\frac{{{E_c}/{E_m} - 1}}{{\exp ({n_x}) - 1}}} \right) \left( {\exp ({n_x}x_r^*) - 1} \right) + 1} \right] \end{aligned}$$

43 $$\begin{aligned} \begin{aligned} D_{11r}^* =&\frac{ - 12b_r^* (h_r^*)^3 \exp \left( - \frac{n_y}{2} \right) }{4n_y^3} \left( 4n_y - 8\exp (n_y) - n_y^2\exp (n_y) \right. \left. + 4n_y\exp (n_y) + n_y^2 + 8 \right) \\&\times \left[ \left( \frac{E_c / E_m - 1}{\exp (n_x) - 1} \right) \left( \exp (n_x x_r^*) - 1 \right) + 1 \right] \end{aligned} \end{aligned}$$

Here,$A_{11r}^*$, $B_{11r}^*$ and $D_{11r}^*$ is the dimensionless stiffness coefficients, while $b_r^*$ and $h_r^*$ are the dimensionless width and height parameters, respectively.

We can derive the updated formulations of the stiffness coefficients $k_{ij}^l$ by substituting Eq. 40 into Eqs. 27, 28, and 29 respectively:

44 $$\begin{aligned} & {k_{rr}^{l} = \frac{{{{(N + 1)}^3}{E_m}{I_0}}}{{{L^3}}}\left( {C_r^* + 4C_{r + 1}^* + C_{r + 2}^*} \right) = \frac{{{{(N + 1)}^3}{E_m}{I_0}}}{{{L^3}}}k_{rr}^{l*},\quad r = 1,...,N} \end{aligned}$$

45 $$\begin{aligned} & {k_{r(r - 1)}^{l} = - \frac{{2{{(N + 1)}^3}{E_m}{I_0}}}{{{L^3}}}\left( {C_r^* + 4C_{r + 1}^*} \right) = \frac{{{{(N + 1)}^3}{E_m}{I_0}}}{{{L^3}}}k_{(r - 1)r}^{l*},\quad r = 2,...,N} \end{aligned}$$

46 $$\begin{aligned} & {k_{r(r - 2)}^{l} = \frac{{{{(N + 1)}^3}{E_m}{I_0}}}{{{L^3}}}C_r^* = \frac{{{{(N + 1)}^3}{E_m}{I_0}}}{{{L^3}}}k_{(r - 2)r}^{l*},\quad r = 3,...,N} \end{aligned}$$

The terms $k_{rr}^{l*}$, $k_{(r-1)r}^{l*}$, and $k_{(r-2)r}^{l*}$ represent the dimensionless components of the linear stiffness tensor. Other components $k_{ij}^{l*}$ are obtained via the symmetry relations from Eqs. 44 to 46, with all other values being zero.

The general formulation of the linear stiffness matrix for the (DPM) can be written as:

47 $$\begin{aligned} \left[ K \right] = \frac{{{{(N + 1)}^3}{E_m}{I_0}}}{{{L^3}}}{\hspace{1.0pt}} \left[ {\begin{array}{*{20}{c}} {k_{11}^{l*}}& {k_{12}^{l*}}& {k_{13}^*}& 0& 0& \ldots & .& \ldots & 0& 0\\ {k_{21}^{l*}}& {k_{22}^{l*}}& {k_{23}^{l*}}& \ddots & 0& \ddots & .& .& .& .\\ {k_{31}^{l*}}& {k_{32}^{l*}}& \ddots & \ddots & \ddots & 0& 0& .& \vdots & \vdots \\ 0& \ddots & \ddots & \ddots & \ddots & \ddots & 0& 0& \vdots & \vdots \\ 0& 0& \ddots & \ddots & \ddots & \ddots & \ddots & \ddots & 0& \vdots \\ .& .& 0& .& .& \ddots & \ddots & \ddots & 0& 0\\ \vdots & .& 0& \ddots & .& \ddots & \ddots & \ddots & \ddots & 0\\ .& \vdots & .& \ddots & 0& \ddots & \ddots & \ddots & \ddots & {k_{N - 2N}^{l*}}\\ .& .& .& .& .& 0& \ddots & \ddots & \ddots & {k_{N - 1N}^{l*}}\\ 0& 0& \ldots & .& .& 0& 0& {k_{NN-2}^{l*}} & {k_{NN - 1}^{l*}}& {k_{NN}^{l*}} \end{array}} \right] \end{aligned}$$

Consequently:

48 $$\begin{aligned} [K] = \frac{{E_m I_0 (N + 1)^3}}{{L^3}} [K^*] \end{aligned}$$

Where $[K^*]$ indicates the dimensionless stiffness matrix pertaining to the discrete system.

####  The stiffness tensor $k^f_{ij}$

The stiffness tensor for the elastic foundation is defined as^48^:

49 $$\begin{aligned} k_{ij}^f =l k_i^{sf} \delta _{ij} = \frac{k_i^{sf*} E_m I_0}{(N + 1) L^3} \delta _{ij} \quad \text {for} \quad i,j = 1, \ldots , N \end{aligned}$$

With $k_r^{sf*}$ taking the values $\eta (1 - \alpha x_r^*)^2$ for ($s=1$) or $\eta (1 + \sin (\alpha x_r^*))$ for ( $s=2$).

The corresponding stiffness matrix for the elastic foundation is given by:

50 $$\begin{aligned} [K^f] = \frac{E_m I_0 (N+1)^3}{L^3} [K^{f*}] \end{aligned}$$

$[K^{f*}]$ is the dimensionless stiffness matrix of the foundation for the (DPM)

51 $$\begin{aligned} \left[ {K_{}^{f*}} \right] = \left[ {\begin{array}{*{20}{c}} {\frac{{k_1^{sf*}}}{{{{(N + 1)}^4}}}}& 0& 0& 0& 0& \ldots & .& \ldots & 0& 0\\ 0& \ddots & 0& 0& 0& \ddots & .& .& .& .\\ 0& 0& \ddots & 0& 0& 0& 0& .& \vdots & \vdots \\ 0& 0& 0& \ddots & \ddots & \ddots & 0& 0& \vdots & \vdots \\ 0& 0& 0& \ddots & \ddots & \ddots & \ddots & \ddots & 0& \vdots \\ .& .& 0& .& .& {\frac{{k_r^{sf*}}}{{{{(N + 1)}^4}}}}& \ddots & \ddots & 0& 0\\ \vdots & .& 0& \ddots & .& \ddots & \ddots & \ddots & \ddots & 0\\ .& \vdots & .& \ddots & 0& \ddots & \ddots & \ddots & \ddots & 0\\ .& .& .& .& .& 0& \ddots & \ddots & \ddots & 0\\ 0& 0& \ldots & .& .& 0& 0& 0& 0& {\frac{{k_N^{sf*}}}{{{{(N + 1)}^4}}}} \end{array}} \right] \end{aligned}$$

In summary, the dimensionless relationships between the dynamic properties of the continuous beam and those of the equivalent (DPM) are as follows: the stiffness of the spiral spring is $C_r^* = D_{11r}^* - {(B_{11r}^*)^2}/{A_{11r}^*}$, the mass is $m_r^* = I_{0r}^*$, and the foundation stiffness is ${k_r^{sf*}}/{(N + 1)^4}$.

### Expression of Natural Vibration Frequency

In this section, we seek to establish the natural frequencies and mode shapes of the discrete physical model (DPM). By applying Eqs. 21, 48, and 50, we formulate the algebraic equation that defines the free vibration characteristics of the DPM.

52 $$\begin{aligned} \left( [K^*] - \omega _{sys}^2 \frac{S_0 \rho _m L^4}{E_m I_0 (N + 1)^4} [M^*] \right) \{ A \} = 0 \end{aligned}$$

The equation can be rewritten in the standard form for eigenvalue problems:

53 $$\begin{aligned} \left( [K^*] - \Omega ^* [M^*] \right) \{ A \} = 0 \end{aligned}$$

Here, $\Omega ^*$ denotes the eigenvalue, while $\{ A \}$ represents the corresponding eigenvector. These values are determined by solving Eq. 53 with the reduced dimensionless mass and stiffness matrices, which are influenced by the boundary conditions.

The natural frequency of the $r^{\text {th}}$ vibrational mode in the discrete system can be computed with the following equation:

54 $$\begin{aligned} \omega _{sys\,r} = \sqrt{\frac{E_m I_0}{\rho _m S_0 L^4}} (N + 1)^2 \sqrt{\Omega _r^*} \quad \text {for} \quad r = 1, \ldots , N \end{aligned}$$

The dimensionless frequency is determined by :

55 $$\begin{aligned} \omega _{sys\,r}^* = \omega _{sys\,r} L^2 \sqrt{\frac{\rho _m S_0}{E_m I_0}} \quad \text {for} \quad r = 1, \ldots , N \end{aligned}$$

Inserting Eq. 54 into Eq. 55 yields the following expression for the dimensionless frequency of the discrete physical model (DPM).

56 $$\begin{aligned} \omega _{sys\,r}^* = (N + 1)^2 \sqrt{\Omega _r^*} \quad \text {for} \quad r = 1, \ldots , N \end{aligned}$$

To ensure convergence and stability, it is recommended that the discretization method maintains a degree of freedom value of at least 100.

## Validation of results

Consider a numerical example of a non-uniform functionally graded material (FGM) beam. The natural frequency is normalised for this comparison as follows :

57 $$\begin{aligned} \omega _{\textrm{sys}}^* = \omega _{\textrm{sys}} L^2 \sqrt{\frac{\rho _0 S_0}{E_0 I_0}} \end{aligned}$$

In this formulation, *L* represents the length of the beam, while $\omega ^*_{\textrm{sys}}$ denotes the dimensionless frequency parameter. The parameters $I_0$, $S_0$, $\rho _0$, and $E_0$ refer to the geometric and material properties at the section located at $x = 0$.

To address this problem, a discrete physical model (DPM) is employed. In the current study, the number of masses *N* was varied between 40 and 200 to evaluate its impact on the accuracy of the vibrational analysis. It was observed that when $N = 100$, the results stabilised and did not change significantly with further increases in *N*. Therefore, $N = 100$ is considered the optimal number of masses for this model.

This indicates that $N = 100$ strikes a balance between computational efficiency and result accuracy. A lower number of masses ($N < 100$) led to less accurate results, while increasing *N* beyond 100 did not improve accuracy. As a result, $N = 100$ is deemed sufficient to ensure convergence and accuracy in this study. This finding aligns with the general principle that a sufficient number of masses are needed to capture the system’s dynamics accurately, without unnecessarily increasing computational effort.

For the calculation of dimensionless frequencies, the number of degrees of freedom (N-DOF) is set to 100 in the MATLAB code. The results from this study are compared with those from^24^ , who investigated a non-uniform axially functionally graded (AFG) simply supported (SS) beam on a variable elastic foundation, and from^18^, who examined a uniform exponentially functionally graded material (FGM) beam. In this case, the material properties for the FGM beam are described by Eqs. 1 and 2, with $n_y = 0$.

### Comparative study 1

This comparison applies to a (SS) beam resting on partial foundations. The stiffness magnitude of the foundations is given by $k_r^{2f*} = \eta (1 + \sin (\alpha x_r^*))$, where $x_r^* = {r}/{(N+1)}$ for $r = 1, \ldots , (N+1)\chi$, with $\chi$ representing the reduced length or position of the foundation. The rotational spring stiffness is $C_r^* = (1 - 0.5 x_r^*)^2$ for $r = 2, \ldots , N+1$, and the additional mass is $m_r^* = 1$. In this case, $C_{1}$ and $C_{N+2}$ are set to zero.

The results are presented in Tables 1 and 2 for various foundation locations under simply supported (SS) boundary conditions. The frequencies obtained from the (DPM) are highly consistent with those derived using the Hencky bar-chain model, as presented in^24^.

The results for boundary conditions (CC), (CS), and (CF) are not covered in^24^. Tables 3, 4, and 5 illustrate the impact of parameters such as foundation intensity $\eta$, location $\chi$, and $\alpha$ on the non-dimensional frequencies for these boundary conditions.

**Fig. 5:**
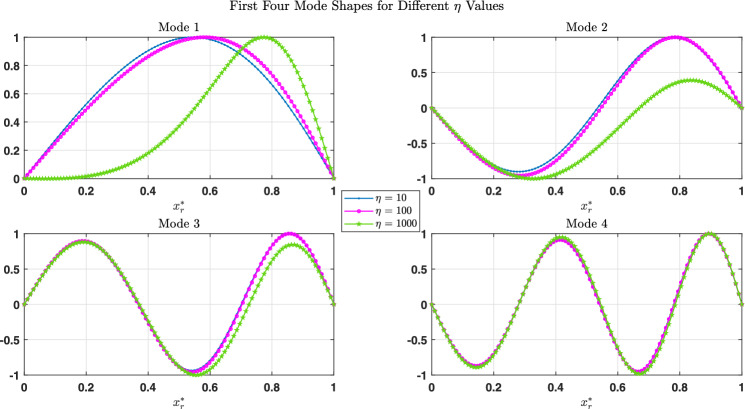
The first four linear mode shapes of a (SS) beam subjected to a partially variable foundation $k_r^{2f*} = \eta (1 + \sin (\alpha x_r^*))$, analyzed for various values of $\eta$, with $\alpha = 3$ and $\chi = 0.7$.

Figure 5 presents the first four normalized mode shapes of the simply supported (SS) beam for $\alpha = 3$ and $\chi = 0.7$ for three values of the foundation stiffness, $\eta = 10$, 100 and 1000. For the first mode, the influence of $\eta$ is marked : as the foundation becomes stiffer, the deformation is progressively shifted and concentrated towards the right part of the span, while the response near the left end is strongly reduced. The second mode also exhibits a noticeable sensitivity to $\eta$, with clear changes in the relative amplitudes of its positive and negative lobes. In contrast, the third and fourth modes are almost unchanged when $\eta$ increases, indicating that the higher modes are only weakly affected by the foundation stiffness, whereas the fundamental mode is the most sensitive to the support conditions.

**Table 1:** Comparison of the fundamental frequency parameters of a (SS) beam partially resting on a variable foundation, described by $k_r^{2f*} = \eta (1 + \sin (\alpha x_r^*))$, for various values of $\alpha$ and $\chi$, with $\eta = 10$.

$\alpha$	$\chi = 0.3$		$\chi = 0.7$		$\chi = 0.4$
	^24^	Present Study	^24^	Present Study	Present Study
1	7.324	7.327	7.998	7.981	7.444
2	—	7.339	—	8.163	7.488
3	7.356	7.351	8.224	8.222	7.522
4	—	7.365	—	8.177	7.543
5	—	7.374	—	8.041	7.551
6	7.381	7.382	7.864	7.863	7.544
7	—	7.379	—	7.697	7.524

**Table 2:** Comparison of the fundamental frequency parameters of a (SS) beam partially resting on a variable foundation, described by $k_r^{2f*} = \eta (1 + \sin (\alpha x_r^*))$, for various values of $\alpha$ and $\chi$, with $\eta = 100$.

$\alpha$	$\chi = 0.3$		$\chi = 0.7$		$\chi = 0.4$
	^24^	Present Study	^24^	Present Study	Present Study
1	8.142	8.136	12.99	13.012	9.154
3	8.397	8.404	14.28	14.303	9.714
6	8.586	8.596	11.98	11.949	9.820

**Table 3:** The fundamental frequency parameters of a (CC) beam on a variable foundation $k_r^{2f*} = \eta (1 + \sin (\alpha x_r^*))$, analyzed for different values of $\eta$, $\chi$, and $\alpha$.

$\eta$	$\alpha$	$\chi = 0.1$	$\chi = 0.3$	$\chi = 0.5$	$\chi = 0.8$
10	1	16.5259	16.5475	16.6954	16.9596
	3	16.5259	16.5548	16.7580	17.0783
	6	16.5260	16.5600	16.7228	16.8046
100	1	16.5271	16.7377	18.1100	20.4531
	3	16.5273	16.8073	18.6442	21.4154
	6	16.5276	16.8556	18.3335	19.0793
1000	1	16.5389	18.2532	26.3011	41.4865
	3	16.5409	18.6923	28.1203	45.9428
	6	16.5437	18.9617	26.6855	31.6554

**Table 4:** The fundamental frequency parameters of a (CS) beam on a variable foundation $k_r^{2f*} = \eta (1 + \sin (\alpha x_r^*))$, analyzed for different values of $\eta$, $\chi$, and $\alpha$.

$\eta$	$\alpha$	$\chi = 0.1$	$\chi = 0.3$	$\chi = 0.5$	$\chi = 0.8$
10	1	11.8304	11.8476	11.9900	12.3961
	3	11.8304	11.8535	12.0486	12.5289
	6	11.8304	11.8576	12.0118	12.1159
100	1	11.8312	11.9988	13.2767	16.6340
	3	11.8314	12.0540	13.7435	17.5816
	6	11.8316	12.0918	13.4395	14.3370
1000	1	11.8398	13.1581	19.5162	38.2187
	3	11.8412	13.4814	20.6969	41.1130
	6	11.8433	13.6745	19.6366	24.4428

**Table 5:** The fundamental frequency parameters of a (CF) beam on a variable foundation $k_r^{2f*} = \eta (1 + \sin (\alpha x_r^*))$, analyzed for different values of $\eta$, $\chi$, and $\alpha$.

$\eta$	$\alpha$	$\chi = 0.1$	$\chi = 0.3$	$\chi = 0.5$	$\chi = 0.8$
10	1	3.1774	3.1840	3.2718	4.0094
	3	3.1774	3.1863	3.3054	4.1206
	6	3.1774	3.1879	3.2794	3.4008
100	1	3.1776	3.2412	3.9111	7.7693
	3	3.1777	3.2617	4.1019	8.0719
	6	3.1777	3.2753	3.9450	4.7156
1000	1	3.1801	3.6218	5.6266	15.2618
	3	3.1805	3.7144	5.8495	15.4635
	6	3.1811	3.7654	5.5866	7.8357

### Comparative study 2

In this section, a comparative analysis is performed on the dimensionless frequencies $w^* = w L^2 \sqrt{{(E_m I_0)}/{(r_m S_0)}}$ with the data presented in^18^ across various boundary conditions. The analysis focuses on a uniform beam characterized by $h^*_r = 1$ and $b^*_r = 1$, which is not influenced by any foundation ($\eta = 0$). Parameters for mass $m^*_r$ and the coefficient $C^*_r$ are calculated using Eqs. 22 and 40 with $n_y = 0$, representing an axial functional gradation (AFG). The results are summarized in Table 6, which highlights the first six dimensionless frequencies of the (AFG) beam under different boundary conditions, with $n_x = 3$. These frequencies are compared to resonance frequencies obtained using the finite element method (FEM) as reported in^18^. The findings demonstrate a strong correlation between the proposed model and the FEM results, regardless of the boundary conditions, indicating that the current discrete model accurately estimates the vibrational modes of the analyzed beam.

**Table 6:** Comparison of the first six dimensionless natural frequencies under different boundary conditions, with no foundation ($\eta = 0$), for $n_x = 3$ and $n_y = 0$.

	C-F		S-S		C-P		C-C	
	DPM	FEM^18^	DPM	FEM^18^	DPM	FEM^18^	DPM	FEM^18^
$\omega _{sys\,1}^*$	2.889	2.852	10.366	10.368	15.739	15.718	24.958	24.942
$\omega _{sys\,2}^*$	21.502	21.494	41.954	41.973	52.860	52.807	67.132	67.113
$\omega _{sys\,3}^*$	3.668	63.673	94.506	94.510	110.670	110.611	130.207	130.236
$\omega _{sys\,4}^*$	126.562	126.575	167.987	167.997	189.345	189.356	214.071	214.258
$\omega _{sys\,5}^*$	211.161	—	261.949	—	288.819	—	318.687	—
$\omega _{sys\,6}^*$	316.642	—	376.840	—	409.004	—	443.963	—

### Special case of a non-uniform exponentially AFG Beam on an elastic foundation

This section examines a specific case involving a non-uniform axially functionally graded (AFG) beam with an exponentially tapered profile, resting on an elastic foundation. The beam has a variable height defined by $h(x) = h_0 \exp (-0.1x/L)$ and a constant width $b(x)=b_0$. Consequently, the non-dimensional mass magnitude is $m_r^* = \exp (-0.4x_r^*)$, and the non-dimensional flexibility of the beam is $C_r^* = \exp (-0.4x_r^*)$.

The beam is subjected to various boundary conditions, including clamped-clamped (CC), clamped-simply supported (CS), simply supported-simply supported (SS), and clamped-free (CF).

Tables 7 and 8 present the first three dimensionless frequencies corresponding to these boundary conditions for two values of $\chi$. Table 7 corresponds to a partial foundation with $\chi =0.5$, while Table 8 corresponds to a total foundation with $\chi =1$. The foundation is of the form $k^{2f*}(x) = \eta (1 + \sin (\alpha x_r^*))$ with $\alpha =3$ and $\eta =100$.

**Table 7:** Dimensionless frequencies of an exponentially tapered beam under various boundary conditions, with a foundation $k_r^{2f*} = \eta (1 + \sin (\alpha x_r^*))$, for $\chi = 0.5$, $\alpha = 3$, and $\eta = 100$.

	Boundary Conditions			
	SS	CC	CS	CF
$\omega ^{*}_{sys\,1}$	14.1442	24.8948	18.0375	15.1451
$\omega ^{*}_{sys\,2}$	40.6229	63.0537	51.6969	25.0844
$\omega ^{*}_{sys\,3}$	89.2546	122.4105	105.4847	63.8664

**Table 8:** Dimensionless frequencies of an exponentially tapered beam under various boundary conditions, with a foundation $k_r^{2f*} = \eta (1 + \sin (\alpha x_r^*))$, for $\chi = 1$, $\alpha = 3$, and $\eta = 100$.

	Boundary Conditions			
	SS	CC	CS	CF
$\omega ^{*}_{sys\,1}$	18.0354	27.2254	22.1144	15.2818
$\omega ^{*}_{sys\,2}$	42.0323	64.0365	52.6770	27.4115
$\omega ^{*}_{sys\,3}$	89.9124	122.8772	106.0800	64.7203

Tables 9 and 10 present the first three dimensionless frequencies of the previously mentioned beam, which is supported by a parabolic foundation described by the equation $k^{1f*}(x) = \eta (1 - \alpha x_r^*)^2$, with $\alpha =3$ and $\eta =100$.

**Table 9:** Dimensionless frequencies of an exponentially tapered beam under various boundary conditions, with a foundation $k_r^{1f*} = \eta (1 - \alpha x_r^*)^2$, for $\chi = 0.5$, $\alpha = 3$, and $\eta = 100$.

	Boundary Conditions			
	SS	CC	CS	CF
$\omega ^{*}_{sys\,1}$	10.1273	22.7047	16.0178	14.0829
$\omega ^{*}_{sys\,2}$	39.5429	62.2943	50.6275	23.1727
$\omega ^{*}_{sys\,3}$	88.8147	122.0557	105.1146	63.1179

**Table 10:** Dimensionless frequencies of an exponentially tapered beam under various boundary conditions, with a foundation $k_r^{1f*} = \eta (1 - \alpha x_r^*)^2$, for $\chi = 1$, $\alpha = 3$, and $\eta = 100$.

	Boundary Conditions			
	SS	CC	CS	CF
$\omega ^{*}_{sys\,1}$	12.7099	27.2254	18.5953	16.3493
$\omega ^{*}_{sys\,2}$	40.9693	64.0365	51.7489	27.3705
$\omega ^{*}_{sys\,3}$	89.4727	122.4823	105.6806	64.2726

For $\chi = 0.5$ (Table 7), the first mode frequency ($\omega ^{*}_{sys\,1}$) is highest under the (CC) boundary condition (24.8948), indicating that clamped ends provide greater stiffness and result in higher frequencies. Conversely, the (SS) boundary condition yields the lowest frequency (14.1442), reflecting increased flexibility. The higher mode frequencies show significant increases, with the third mode ($\omega ^{*}_{sys\,3}$) reaching 122.4105 for the (CC) condition.

When $\chi = 1$ (Table 8), the first mode frequencies increase across all boundary conditions compared to $\chi = 0.5$, with the CC boundary condition again exhibiting the highest frequency (27.2254). This increase suggests that a fully supported foundation enhances stiffness, leading to higher frequencies. The trend observed in the higher modes is consistent with the partial foundation case, with all frequencies increasing.

For $\chi = 0.5$ (Table 9), the first mode frequencies are lower across all boundary conditions compared to those in Tables 7 and 8, with the highest value under the CC condition (22.7047). This reduction in frequency is due to the parabolic foundation, which provides less stiffness compared to the sinusoidal foundation.

When $\chi = 1$ (Table 10), the frequencies increase again for all boundary conditions, with the (CC) boundary condition yielding the highest frequency (27.2254). The parabolic foundation continues to produce lower frequencies than the sinusoidal foundation, demonstrating that the type of foundation significantly impacts the vibrational characteristics.

## Numerical results and discussions

Upon validation of the proposed model, it will be employed to investigate the free vibration characteristics of non-uniform (2D-FGM) beams featuring diverse profiles and boundary conditions in the presence of a foundation.

### Uniform (2D-FGM ) beam without elastic foundation

This section focuses on computing the dimensionless frequencies for a uniform beam made from a two-dimensional functionally graded material (2D-FGM), with $h_r^* = b_r^* = 1$, under various gradient indices ($n_x$ and $n_y$). The beam is subjected to different boundary conditions, including clamped-free (CF), clamped-clamped (CC), and simply supported-simply supported (SS).

Tables 11, 12, and 13 present the dimensionless frequencies of the first three vibration modes of the beam under the (SS), (CC), and (CF) boundary conditions, respectively. The values of $\omega _r^*$ for $r = 1, 2,$ and 3 are provided for increasing values of $n_x$ and $n_y$.

For all boundary conditions, the frequencies decrease with increasing $n_x$ for a fixed $n_y$. For instance, when $n_y = 0.2$, the frequency drops from 10.82 to 4.63 as $n_x$ increases from 0.2 to 8. Similarly, frequencies also decrease with increasing $n_y$ for a fixed $n_x$. For example, with $n_x = 0.2$, the frequency decreases from 10.82 to 4.63 as $n_y$ increases from 0.2 to 8.

The overall trend indicates that the vibrational frequencies decrease with both $n_x$ and $n_y$. This behavior can be attributed to the increase in the structure’s mass and rigidity. Specifically, as the grading indices $n_x$ and $n_y$ increase, the effective mass of the beam rises, which is similar to adding mass to a spiral spring system. In this analogy, when additional mass is introduced, the stiffness of the spring becomes less effective at resisting displacement, leading to lower vibrational frequencies.

The power indices significantly influence the distribution of material properties, which in turn affects the stiffness characteristics of the beam. As the indices increase, the material transitions toward a configuration that is softer, analogous to a spiral spring that has been stretched. This results in modifications to the beam’s stiffness matrix, where the effective spring constants decrease, further contributing to the reduction in vibrational frequencies.

**Fig. 6:**
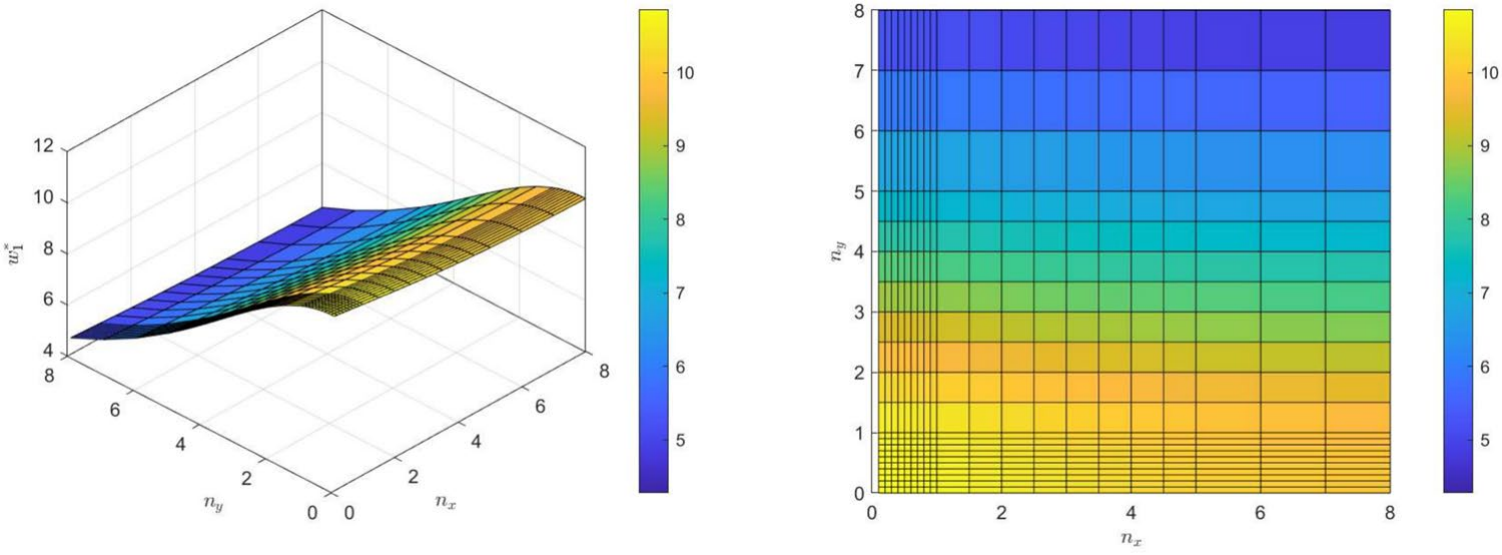
Variation of the first dimensionless frequency parameter of a (SS) beam with gradation indexes $n_x$ and $n_y$.

An interesting observation is that, for a fixed value of $n_y$, a variation in $n_x$ leads to slight frequency variations, whereas for a fixed value of $n_x$, an increase in $n_y$ results in more significant frequency changes, as illustrated in Fig. 6. This suggests that the influence of $n_y$ is more pronounced, possibly due to its role in modulating the vertical stiffness, while $n_x$ affects the horizontal gradient and mass distribution to a lesser extent.

This nuanced response underscores the complexity of the interaction between the beam’s geometric and material properties. Changes in the indices $n_x$ and $n_y$ not only alter the material composition but also significantly modify the stiffness and mass matrices of the system, ultimately impacting the dynamic characteristics of the beam.

**Table 11:** Variation of the first three dimensionless frequency parameters of a uniform (2D-FGM) (SS) beam with gradation indexes $n_x$ and $n_y$.

First Mode for SS								
$n_x$	$n_y$							
	0.2	0.4	0.8	1	2	4	6	8
0.2	10.82	10.78	10.66	10.56	9.85	7.82	5.96	4.63
0.4	10.78	10.75	10.62	10.53	9.82	7.80	5.94	4.62
0.6	10.75	10.71	10.59	10.50	9.79	7.77	5.92	4.60
0.8	10.71	10.68	10.55	10.46	9.76	7.75	5.90	4.59
2	10.50	10.47	10.35	10.26	9.57	7.60	5.79	4.50
4	10.22	10.19	10.07	9.98	9.31	7.39	5.64	4.38
8	9.97	9.94	9.82	9.73	9.08	7.21	5.49	4.27

Second Mode for SS								
$n_x$	$n_y$							
	0.2	0.4	0.8	1	2	4	6	8
0.2	43.49	43.36	42.84	42.47	39.60	31.45	23.98	18.64
0.4	43.38	43.25	42.74	42.36	39.50	31.37	23.92	18.60
0.6	43.26	43.13	42.63	42.25	39.40	31.29	23.85	18.55
0.8	43.15	43.02	42.51	42.14	39.30	31.20	23.79	18.50
2	42.45	42.33	41.83	41.46	38.66	30.70	23.41	18.20
4	41.42	41.29	40.81	40.45	37.72	29.95	22.84	17.76
8	40.27	40.15	39.68	39.33	36.68	29.12	22.20	17.26

Third Mode for SS								
$n_x$	$n_y$							
	0.2	0.4	0.8	1	2	4	6	8
0.2	97.66	97.37	96.22	95.38	88.95	70.63	53.85	41.87
0.4	97.42	97.13	95.99	95.15	88.73	70.45	53.72	41.77
0.6	97.18	96.89	95.74	94.91	88.50	70.27	53.58	41.66
0.8	96.93	96.64	95.50	94.66	88.28	70.09	53.44	41.56
2	95.44	95.15	94.03	93.21	86.92	69.02	52.62	40.92
4	93.31	93.03	91.94	91.13	84.98	67.48	51.45	40.01
8	90.93	90.66	89.59	88.80	82.81	65.76	50.14	38.98

**Table 12:** Variation of the first three dimensionless frequency parameters of a uniform (2D-FGM) (CC) beam with gradation indexes $n_x$ and $n_y$.

First Mode for CC								
$n_x$	$n_y$							
	0.2	0.4	0.8	1	2	4	6	8
0.2	24.69	24.62	24.33	24.12	22.49	17.86	13.61	10.58
0.4	24.72	24.65	24.36	24.15	22.52	17.88	13.63	10.60
0.6	24.76	24.68	24.39	24.18	22.55	17.90	13.65	10.61
0.8	24.79	24.72	24.43	24.21	22.58	17.93	13.67	10.63
2	24.98	24.90	24.61	24.40	22.75	18.06	13.77	10.71
4	25.17	25.10	24.80	24.59	22.93	18.20	13.88	10.79
8	25.08	25.01	24.71	24.50	22.84	18.14	13.83	10.75

Second Mode for CC								
$n_x$	$n_y$							
	0.2	0.4	0.8	1	2	4	6	8
0.2	68.29	68.09	67.28	66.69	62.19	49.38	37.65	29.28
0.4	68.24	68.04	67.23	66.65	62.15	49.35	37.63	29.26
0.6	68.19	67.99	67.18	66.60	62.10	49.31	37.60	29.23
0.8	68.14	67.93	67.13	66.54	62.06	49.27	37.57	29.21
2	67.80	67.59	66.80	66.21	61.75	49.03	37.38	29.07
4	67.27	67.07	66.28	65.70	61.27	48.65	37.09	28.84
8	66.70	66.50	65.71	65.14	60.75	48.23	36.78	28.59

Third Mode for CC								
$n_x$	$n_y$							
	0.2	0.4	0.8	1	2	4	6	8
0.2	133.98	133.58	132.01	130.85	122.02	96.86	73.88	57.44
0.4	133.77	133.37	131.79	130.64	121.83	96.74	73.76	57.35
0.6	133.55	133.15	131.58	130.43	121.63	96.58	73.64	57.26
0.8	133.32	132.92	131.36	130.21	121.42	96.41	73.51	57.16
2	131.96	131.57	130.02	128.88	120.18	95.43	72.76	56.58
4	130.06	129.67	128.15	127.02	118.45	94.06	71.72	55.76
8	128.37	127.99	126.48	125.37	116.92	92.83	70.78	55.04

**Table 13:** Variation of the first three dimensionless frequency parameters of a uniform (2D-FGM) (CF) beam with gradation indexes $n_x$ and $n_y$.

First Mode for CF								
$n_x$	$n_y$							
	0.2	0.4	0.8	1	2	4	6	8
0.2	2.94	2.93	2.89	2.87	2.67	2.12	1.62	1.26
0.4	2.93	2.92	2.88	2.86	2.66	2.11	1.61	1.25
0.6	2.92	2.91	2.87	2.85	2.65	2.11	1.61	1.25
0.8	2.91	2.90	2.86	2.84	2.65	2.10	1.60	1.24
2	2.87	2.86	2.83	2.80	2.62	2.08	1.58	1.23
4	2.88	2.87	2.84	2.81	2.62	2.08	1.59	1.23
8	3.00	3.00	2.96	2.93	2.74	2.17	1.65	1.29

Second Mode for CF								
$n_x$	$n_y$							
	0.2	0.4	0.8	1	2	4	6	8
0.2	22.47	22.41	22.14	21.95	20.47	16.25	12.39	9.63
0.4	22.41	22.34	22.07	21.88	20.41	16.20	12.35	9.60
0.6	22.34	22.27	22.01	21.81	20.34	16.15	12.31	9.57
0.8	22.27	22.20	21.94	21.75	20.28	16.10	12.28	9.55
2	21.87	21.80	21.55	21.36	19.92	15.81	12.06	9.37
4	21.33	21.27	21.02	20.83	19.43	15.42	11.76	9.14
8	20.86	20.80	20.55	20.37	19.00	15.08	11.50	8.94

Third Mode for CF								
$n_x$	$n_y$							
	0.2	0.4	0.8	1	2	4	6	8
0.2	66.52	66.32	66.54	66.52	66.52	66.52	66.52	66.52
0.4	66.35	66.15	66.37	66.35	66.35	66.35	66.35	66.35
0.6	66.17	65.97	66.19	66.17	66.17	66.17	66.17	66.17
0.8	65.99	65.79	65.61	65.99	65.99	65.99	65.99	65.99
2	64.81	64.62	64.86	64.81	64.81	64.81	64.81	64.81
4	62.96	62.77	62.03	62.96	62.96	62.96	62.96	62.96
8	60.77	60.59	60.88	60.77	60.77	60.77	60.77	60.77

### Non-uniform (2D-FGM) without any foundation

This subsection presents the vibrational behavior of a (2D-FGM) tapered beam, with mechanical properties defined previously in Eqs. 1 and 2. Various parameters, such as the index parameters ($n_x$, $n_y$, and $\gamma$) for material distribution and non-uniformity, are considered.

**Fig. 7:**
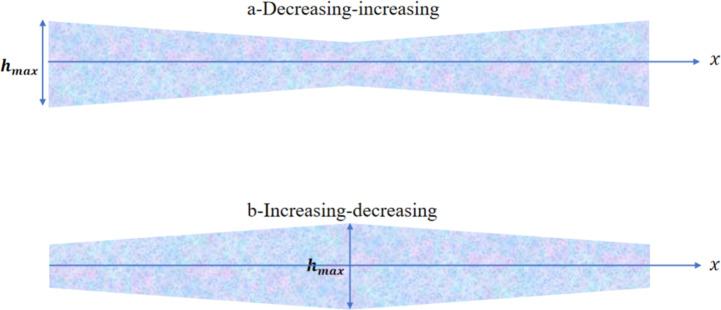
The profiles for the non-uniform beam : **a**) (decreasing-increasing), and **b**) (increasing-decreasing).

Several example problems involving non-uniform geometries are examined to demonstrate the simplicity of using (DPM) for analyzing the vibration frequencies of (2D-FGM) beams with a non-uniform profile. Two types of beam profiles are considered, as shown in Fig 7.

The thickness of the beam, *h*(*x*), as a function of *x*, can be expressed using the equations for $h_1(x)$ and $h_2(x)$. The thickness variation (decreasing-increasing), shown in Fig 7-a, is defined by: $h_1(x) = h_{\text {max}} \left[ 1 - \gamma \left( 1 - \left| 1 - \frac{2x}{L} \right| \right) ^n \right] ,$ and the thickness variation (increasing-decreasing), shown in Fig 7-b, is defined by: $h_2(x) = h_{\text {max}} \left[ 1- \gamma \left| 1 - \frac{2x}{L} \right| ^n \right] ,$ with $h_{\text {max}}$ representing the maximum height chosen as the reference height in this study. In the frequency calculation, the non-dimensional height presented in Eqs. 22, 41, 42, and 43 can be given by $h^*_{r1} = \left[ 1 - \gamma \left( 1 - \left| 1 - 2x_r^* \right| \right) ^n \right] ,$ or $h^*_{2r} = \left[ 1- \gamma \left| 1 - 2x_r^* \right| ^n \right] ,$ with $b_r^* = 1$.

**Fig. 8:**
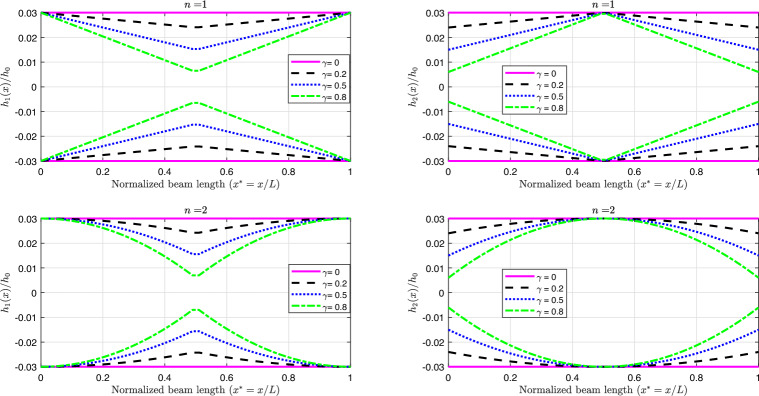
The profiles for the non-uniform beam for various values of $\gamma$.

Each variation in the thickness of the beam can follow either a linear $(n=1)$ or parabolic $(n=2)$ profile, as illustrated in Fig 8. These variations are assumed to be symmetric about the beam’s mid-span (*L*/2).

**Fig. 9:**
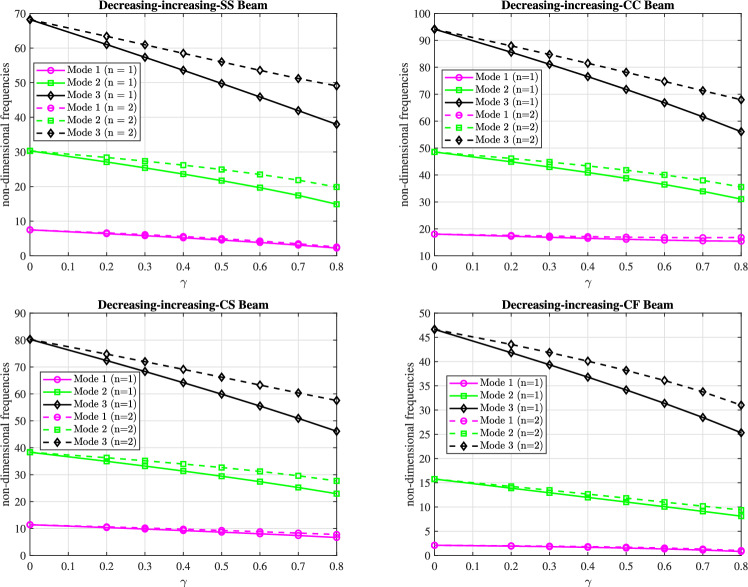
The effect of the parameter $\gamma$ on the first three dimensionless frequencies for the (decreasing-increasing) beam under (SS), (CC), (CS), and (CF) boundary conditions.

**Fig. 10:**
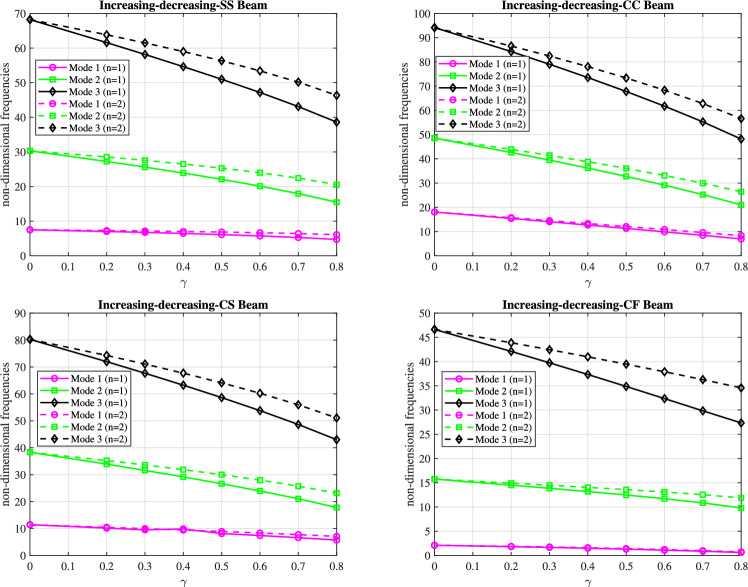
The effect of the parameter $\gamma$ on the first three dimensionless frequencies for the (increasing-decreasing) beam under (SS), (CC), (CS), and (CF) boundary conditions.

The graphs in Figs. 9 and 10 present the first three dimensionless frequencies for $(n_x=3)$ and $(n_y=4)$ as a function of the taper parameter $\gamma$ for the boundary conditions (SS), (CC), (CS), and (CF).

Fig 9 shows the frequencies for a (decreasing-increasing) taper profile, while Fig 10 shows the frequencies for an (increasing-decreasing) taper profile. In both figures, the frequencies generally decrease as $\gamma$ increases, regardless of the boundary conditions or the taper profile (linear with $n=1$ or parabolic with $n=2$). The (CC) boundary condition results in the highest frequencies, indicating the stiffest configuration, while the (CF) boundary condition produces the lowest frequencies due to increased flexibility. The (SS) and (CS) boundary conditions exhibit frequencies that fall between the (CC) and (CF) conditions, with the (CS) beam showing higher frequencies than the (SS) beam but lower than the (CC) beam.

The reduction in frequencies with increasing $\gamma$ can be attributed to the increasing tapering of the beam, which leads to a reduction in the effective stiffness. As the taper parameter $\gamma$ increases, the beam’s profile becomes more pronounced, resulting in a greater mass distribution towards the ends and a less rigid structure overall. This behavior can be interpreted in the context of a conical beam: as the taper increases, the flexibility of the beam can be modeled as $N+2$ interconnected spiral springs, which collectively become less effective at resisting deformation, resulting in lower vibrational frequencies.

For linear and parabolic conicity profiles, beams with parabolic conicity $(n=2)$ exhibit slightly higher frequencies than beams with linear conicity $(n=1)$ in both profiles. This indicates that the parabolic conicity profile significantly affects the dynamic behavior of the beam in both cases. These trends are consistent across two mode shapes (modes 2 and 3). In mode 1, however, the frequency remains virtually constant.

**Fig. 11:**
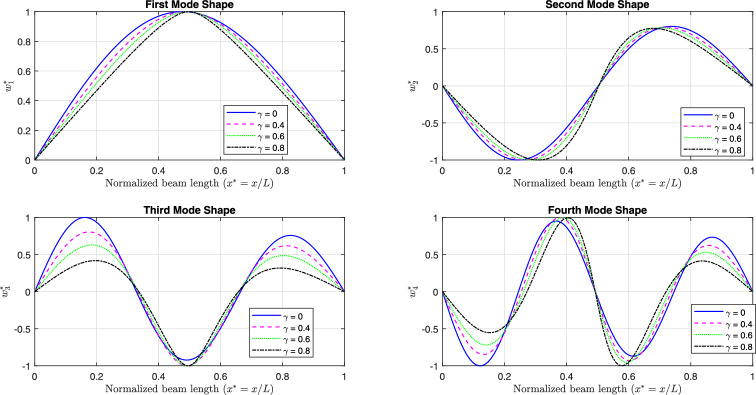
The first four linear mode shapes of a parabolically (decreasing-increasing) (SS) beam for different values of $\gamma$, with $n_x = 3$ and $n_y =4$.

**Fig. 12:**
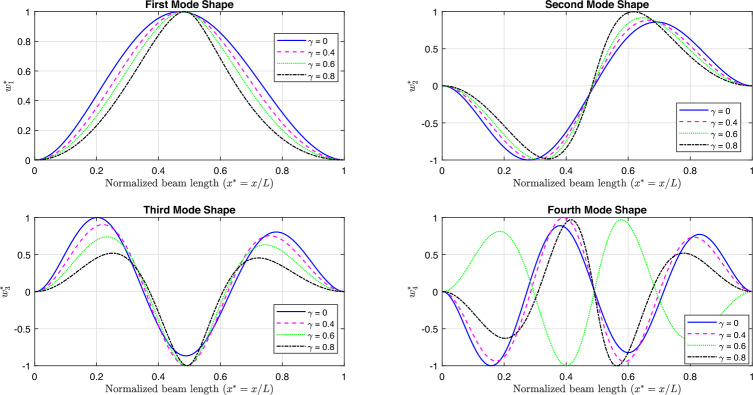
The first four linear mode shapes of a parabolically (decreasing-increasing) (CC) beam for different values of $\gamma$, with $n_x = 3$ and $n_y = 4$.

Fig 11 and Fig 12 illustrate the first four linear mode shapes of a parabolically (decreasing-increasing) simply supported (SS) beam and a clamped-clamped (CC) beam, respectively, for a range of $\gamma$ values. In both figures, $n_x = 3$ and $n_y = 4$.

**Fig. 13:**
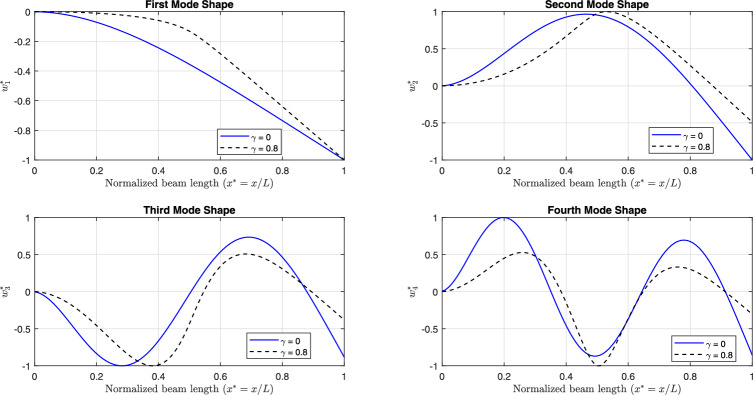
The first four linear mode shapes of a parabolically (decreasing-increasing) (CF) beam for $\gamma =0$ and 0.8, with $n_x = 3$ and $n_y = 4$.

**Fig. 14:**
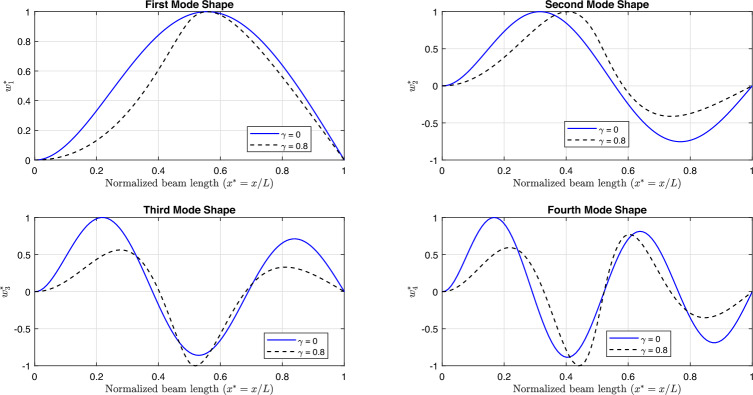
The first four linear mode shapes of a parabolically (decreasing-increasing) (CS) beam for $\gamma =0$ and 0.8, with $n_x = 3$ and $n_y = 4$.

Fig 13 and Fig 14 present the first four linear mode shapes of a parabolically (decreasing-increasing) clamped-free (CF) beam and a clamped-supported (CS) beam, respectively, for $\gamma = 0$ and $\gamma = 0.8$. In both figures,$n_x = 3$ and $n_y = 4$.

### Non-uniform (2D-FGM) Beam under elastic foundation

**Fig. 15:**
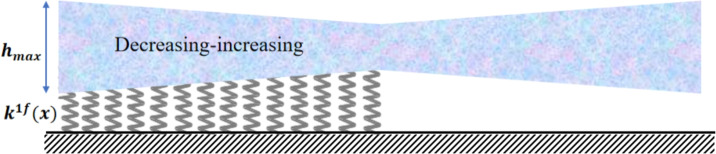
The non-uniform (decreasing-increasing) beam under a partially variable foundation.

This section presents an analysis of the vibration frequencies of a non-uniform (2D-FGM) beam with a height profile described by $h(x) = h_{\text {max}} \left[ 1 - \gamma \left( 1 - \left| 1 - {2x}/{L} \right| \right) ^n \right]$. The beam is supported by a partially elastic foundation with variable stiffness, characterized by $k^{1f*}(x) = \eta (1 + \alpha \sin (x))$, Fig 15.

**Fig. 16:**
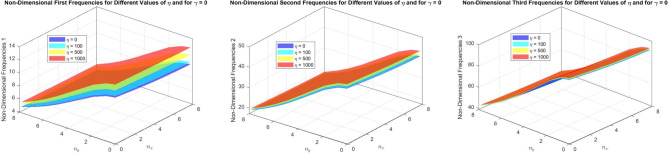
The effects of the parameter $\eta$ on the first three dimensionless frequencies for a (SS) parabolically (decreasing-increasing) beam (FTDISSB) with $\chi = 0.2$ and $\gamma = 0$.

**Fig. 17:**
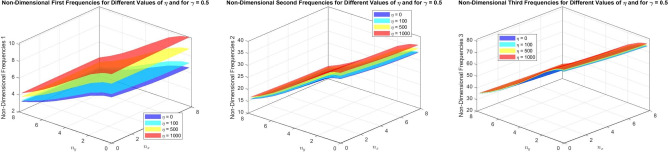
The effects of the parameter $\eta$ on the (FTDISSB) with $\chi = 0.2$ and $\gamma = 0.5$.

**Fig. 18:**
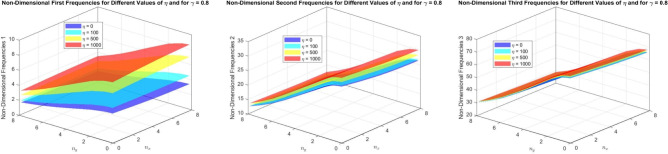
The effects of the parameter $\eta$ on the (FTDISSB) with $\chi = 0.2$ and $\gamma = 0.8$.

**Fig. 19:**
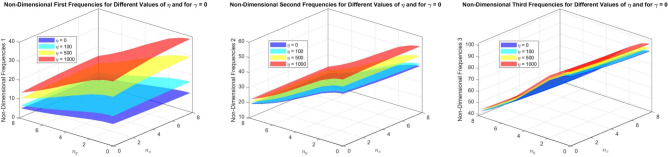
The effects of the parameter $\eta$ on the (FTDISSB) with $\chi = 0.7$ and $\gamma = 0$.

**Fig. 20:**
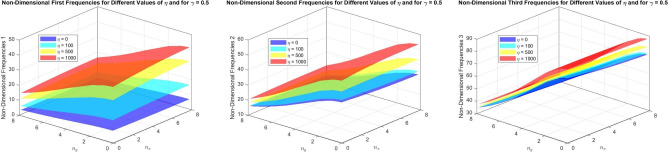
The effects of the parameter $\eta$ on the (FTDISSB) with $\chi = 0.7$ and $\gamma = 0.5$.

**Fig. 21:**
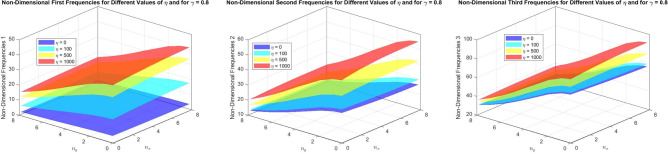
The effects of the parameter $\eta$ on the (FTDISSB) with $\chi = 0.7$ and $\gamma = 0.8$.

Figures 16 to 21 present the first three dimensionless frequencies for the following simulation parameters: varying values of the foundation stiffness parameter $\eta = 0, 100, 500, 1000$, different values of $n_x$ and $n_y$, with $\alpha = 3$ and $n = 2$ for a taper $\gamma$ of 0, 0.5,  and 0.8. The beam is analyzed with the foundation over the interval $x = 0$ to $\chi (N + 1)$.

For Figs. 16 to 18, $\chi$ is set to 0.2.

In Fig. 16, where $\gamma = 0$, it is observed that as $\eta$ increases, the frequencies for the second and third modes remain virtually constant compared to the first frequencies, which increase with $\eta$. The mode shapes remain relatively stable, indicating that the uniform cone maintains a stable mode shape profile for the second and third frequencies.

The observation can be explained by the fact that the first mode is more sensitive to changes in the foundation stiffness $\eta$ than the higher modes. As $\eta$ increases, the first mode frequency rises, reflecting the beam’s stiffness due to stronger foundation support. However, the variation in $\eta$ has little effect on the second and third mode frequencies. This suggests that the mode shapes for these higher frequencies are more dependent on the beam’s geometric and material properties rather than the foundation stiffness.

Fig. 17, where $\gamma = (0.5)$, the increase in $\eta$ also leads to higher frequencies. The slope of the cone ($\gamma = 0.5$) affects the frequencies, showing a slight variation compared to the non-conical beam. The mode shapes for the first and second modes remain stable, as indicated in the previous figure, but there is a slight modification in the frequency values due to the conical nature of the beam.

Fig. 18, which represents $\gamma = 0.8$, continues to demonstrate that frequencies increase with $\eta$. The conicity ($\gamma = 0.8$) reveals the impact of the foundation on the frequencies compared to lower conicity values. At this level of conicity, the dimensionless frequencies show a decrease relative to $\gamma = 0$ and $\gamma = 0.5$. Conversely, a higher conicity amplifies the effect of the variable stiffness of the foundations, making the influence of the foundations particularly noticeable on the first and second modes.

For Figs. 19 to 21, $\chi$ is set to 0.7, indicating that the foundation is situated within a reduced length of 0.7.

In Fig. 19, where $\gamma = 0$, we observe that as $\eta$ increases, the frequencies for all modes exhibit a notable increase compared to the frequencies observed at $\chi = 0.2$. This suggests that the foundation’s influence is becoming more pronounced at this higher value of $\chi$. The first mode frequency continues to rise, reflecting the increased stiffness of the beam due to enhanced foundation support. However, the most significant change is seen in the second and third mode frequencies, which are now more sensitive to variations in foundation stiffness, indicating a shift in the mode shapes’ dependence on the foundation characteristics.

In Fig. 20, where $\gamma = 0.5$, the trend of increasing frequencies with rising $\eta$ persists. The conical slope of the beam introduces a slight variation in frequency values compared to the non-conical case. Notably, the frequencies for the second and third modes show a more significant increase than those observed in Fig. 17 with $\chi = 0.2$. This indicates that the conical geometry, combined with the higher $\chi$, enhances the sensitivity of these modes to changes in foundation stiffness. The mode shapes remain relatively stable, but the increased frequencies highlight the greater impact of the foundation on the dynamic behavior of the system.

Figure 21, representing $\gamma = 0.8$, further illustrates the increasing trend of frequencies with $\eta$. At this higher conicity, the foundation’s effect on the frequencies becomes particularly pronounced, especially for the second and third modes. Compared to the previous figures, the dimensionless frequencies show a marked increase, indicating a stronger interaction between the conical geometry and the foundation stiffness. The higher conicity amplifies the variable stiffness effect of the foundations, making the influence on the first and second modes especially clear. This reinforces the notion that as $\chi$ increases, the foundation’s role in determining the dynamic characteristics of the system becomes more significant, particularly for higher modes.

**Fig. 22:**
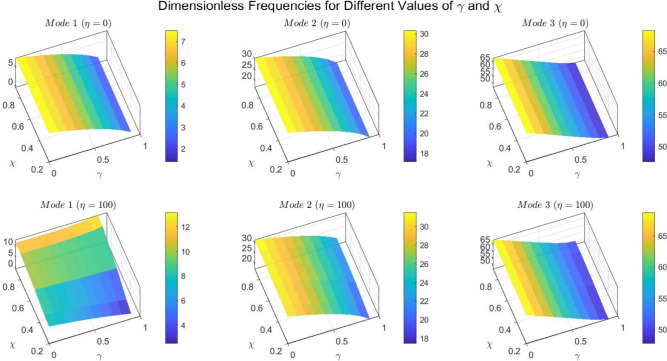
Effects of the parameters $\gamma$ and $\chi$ on dimensionless frequencies for a (SS) non-uniform (decreasing-increasing) (2D-FGM) beam with $n_x = 3$ and $n_y = 4$.

**Fig. 23:**
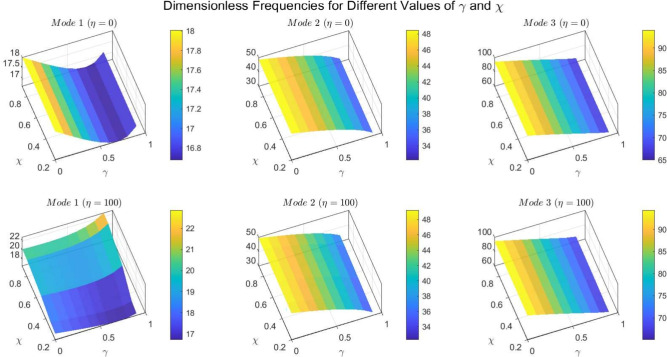
Effects of the parameters $\gamma$ and $\chi$ on dimensionless frequencies for a (CC) non-uniform (decreasing-increasing) (2D-FGM) beam with $n_x = 3$ and $n_y = 4$.

**Fig. 24:**
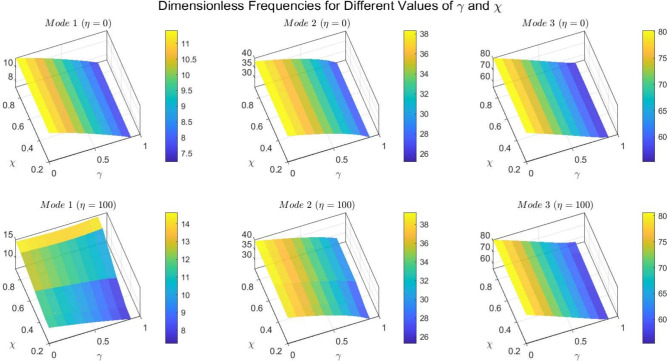
Dimensionless Frequencies for Different Values of $\gamma$ and $\chi$ for a (CS) (decreasing-increasing) (2D-FGM) with $n_x=3$ and $n_y=4$.

**Fig. 25:**
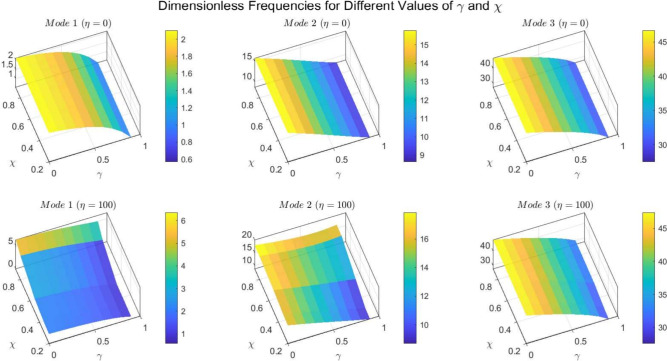
Effects of the parameters $\gamma$ and $\chi$ on dimensionless frequencies for a (CF) non-uniform (decreasing-increasing) (2D-FGM) beam with $n_x = 3$ and $n_y = 4$.

Figures 22 to 25 display the first three dimensionless frequencies in 3D for a (decreasing-increasing) (2D-FGM) beam with $n_x=3$ and $n_y=4$. These figures illustrate the effects of different values of $\gamma$ and $\chi$ on the frequencies for various boundary conditions: (SS), (CC), (CS), and (CF).

From these figures, it is evident that increases in the parameter $\chi$ and the conicity parameter $\gamma$ significantly affect the first vibrational frequency across all boundary conditions. This influence is more pronounced compared to the second and third frequencies. An interesting observation is that the second frequency, which corresponds to the (CF) boundary condition, is also affected by variations in $\gamma$ and the foundation stiffness parameter $\eta$.

Table 14 shows the frequencies for the first three modes of vibration of a parabolic beam with simply supported ends. The frequencies are listed for different values of $\chi$ and $\gamma$, indicating how the beam’s response varies with these parameters. As $\chi$ increases, the frequencies for Mode 1 decrease significantly, while Modes 2 and 3 exhibit a more gradual decline.

In Table 15, the frequencies for the same modes are displayed under clamped boundary conditions. Compared to the SS condition, the frequencies are generally higher across all modes. This indicates that clamping the beam results in stiffer behavior, which raises the natural frequencies. The trend of decreasing frequencies with increasing $\chi$ is consistent with the SS case.

Table 16 illustrates the frequencies for the (CS) beam configuration. The frequencies for Mode 1 are lower than those observed in both the (SS) and (CC) conditions, reflecting the more flexible nature of a (CS) beam. The data shows a similar trend of decreasing frequencies with increasing $\chi$ and varying $\gamma$.

Finally, Table 17 presents the frequencies for a (CF) beam configuration. The frequencies for Mode 1 are the lowest among all boundary conditions, highlighting the significant impact of fixing the beam’s ends on its vibrational characteristics. The decreasing trend with respect to $\chi$ continues, but the absolute values are lower than those in the SS and CC conditions.

**Fig. 26:**
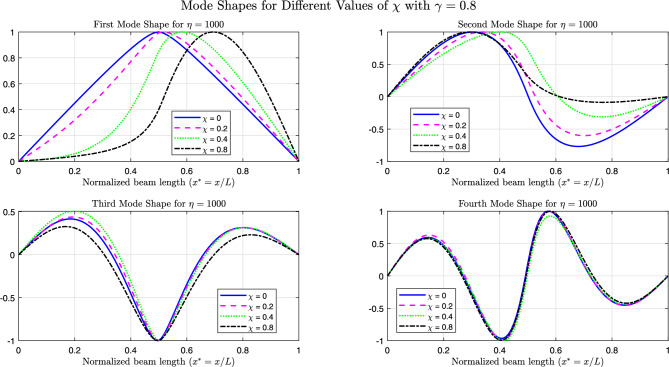
The first four linear mode shapes of a parabolically (decreasing-increasing) (SS) beam for $\gamma =0.8$ and $\eta =1000$, with $n_x = 3$ and $n_y = 4$.

**Fig. 27:**
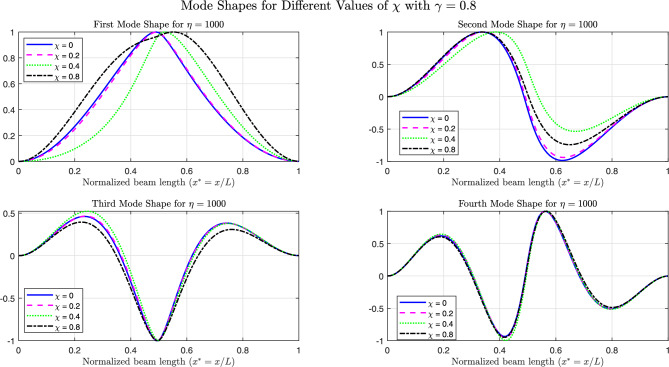
The first four linear mode shapes of a parabolically (decreasing-increasing) (CC) beam for $\gamma =0.8$ and $\eta =1000$, with $n_x = 3$ and $n_y = 4$.

**Fig. 28:**
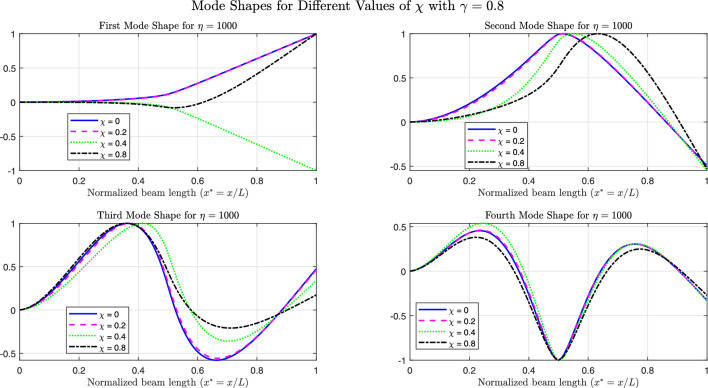
The first four linear mode shapes of a parabolically (decreasing-increasing) (CF) beam for $\gamma =0.8$ and $\eta =1000$, with $n_x = 3$ and $n_y = 4$.

The Figs. 26, 27, and 28 present the first four normalized mode shapes for simply supported (SS), clamped (CC), and cantilever (CF) boundary conditions, respectively. These results are shown for different values of $\chi$ with $\gamma = 0.8$ and $\eta = 1000$.

The numerical results obtained for the non-uniform 2D-FGM beam provide several useful physical insights into the influence of material gradation, geometric tapering and elastic support on the free vibration response. The bending stiffness of the beam is modeled by spiral springs whose stiffness varies according to the gradation indices $n_x$ and $n_y$, while the thickness varies parabolically (decreasing–increasing) with the taper ratio $\gamma$. As a consequence, the central part of the beam is thinner, more flexible and much more sensitive to any additional support than the regions close to the ends. The Winkler foundation is represented by vertical springs characterized by the intensity parameter $\eta$ and the reduced length $\chi$, which denotes the portion of the span actually resting on the foundation.

From a global frequency point of view, increasing $\eta$ stiffens the supported region and produces a marked increase in the fundamental frequency, in particular when the foundation covers the most flexible zone of the beam. This strong sensitivity of the first mode is consistent with its deformation shape: for simply supported (SS) and clamped–free (CF) beams, the first mode is dominated by one large deflected region over the span. When $\chi$ increases, a larger fraction of this deflection lies on the stiff foundation; the supported part then behaves almost like a rigid or fixed region and the deformation is pushed into the remaining free length. This explains the significant changes observed in the first-mode shape and frequency.

In contrast, the higher modes exhibit several internal nodes and alternating deflected regions of opposite sign. Over the supported zone, these patterns tend to balance each other and the net effect of the foundation on the global response is reduced. As a result, the second and third natural frequencies vary only moderately with $\eta$, and the third and fourth mode shapes are only slightly modified, even for large foundation stiffness and long supported lengths.

This interpretation also clarifies the differences between boundary conditions. In the clamped–clamped (CC) case, the beam ends are already very stiff, so the relative increase in stiffness provided by the foundation is smaller: only the first and, to a lesser extent, the second mode show visible modifications, while the higher modes, whose deformation patterns are already strongly constrained by the end fixity, remain almost unchanged. For the CF beam, the first mode is mainly governed by the deflection near the clamped end; when the foundation is located in this highly flexible region, it acts almost as an additional support and the first mode is strongly reshaped, whereas higher modes progressively shift their dominant deformation toward the free end and become less sensitive to the foundation.

The taper ratio $\gamma$ and the gradation indices $n_x$ and $n_y$ further modulate these mechanisms. A stronger taper or a material gradation that reduces stiffness in the middle of the span amplifies the contrast between the central flexible zone and the stiffer regions, so placing a stiff foundation under this zone has a pronounced effect on the first and sometimes the second mode. Conversely, modes whose main deformation is located in already stiff regions, or outside the supported length $\chi$, are much less influenced by changes in $\eta$ and $\chi$. Overall, the results show that the rigid foundation does not act uniformly on all modes : it primarily affects those modes that mobilize large deflections in the supported region, while modes with nodes or small displacements over this region are only marginally influenced. This combined interpretation clarifies why some foundations strongly affect certain modes but not others, and highlights the coupled influence of $\eta$, $\chi$, $\gamma$, $n_x$ and $n_y$ on the dynamic behavior of tapered 2D-FGM beams.

**Table 14:** Variation of the First Three Modal Frequencies for a SS (2D-FGM) Beam with Changes in $\chi$ and $\gamma$, $n_x = 3$, $n_y = 4$, $\eta = 100$, and $n = 2$.

Frequencies for Different Modes under (SS) Boundary Condition					
$\gamma$	$\chi$				
	0.2	0.3	0.5	0.8	0.9
**Mode 1**					
0.0	7.7193	8.2097	9.9405	11.666	11.782
0.2	6.8712	7.4461	9.5287	11.559	11.686
0.3	6.3957	7.0263	9.3319	11.548	11.682
0.4	5.8780	6.5778	9.1472	11.578	11.719
0.5	5.3105	6.0989	8.9816	11.662	11.812
0.6	4.6848	5.5906	8.8434	11.818	11.978
0.7	3.9940	5.0611	8.7406	12.070	12.242
0.8	3.2435	4.5384	8.6679	12.446	12.634
**Mode 2**					
0.0	30.514	30.800	31.062	31.409	31.513
0.2	28.618	28.945	29.274	29.663	29.771
0.3	27.566	27.918	28.292	28.707	28.817
0.4	26.424	26.805	27.237	27.681	27.793
0.5	25.167	25.582	26.091	26.568	26.682
0.6	23.756	24.213	24.829	25.342	25.459
0.7	22.127	22.636	23.411	23.963	24.082
0.8	20.147	20.725	21.764	22.354	22.474
**Mode 3**					
0.0	68.359	68.419	68.549	68.691	68.754
0.2	63.530	63.599	63.777	63.962	64.028
0.3	61.082	61.156	61.368	61.583	61.651
0.4	58.617	58.696	58.955	59.208	59.277
0.5	56.149	56.234	56.558	56.863	56.932
0.6	53.705	53.796	54.214	54.591	54.661
0.7	51.347	51.444	52.007	52.489	52.558
0.8	49.221	49.323	50.130	50.780	50.845

**Table 15:** Variation of the first three modal frequencies for a CC (2D-FGM) Beam with Changes in $\chi$ and $\gamma$, $n_x = 3$, $n_y = 4$, $\eta = 100$, and $n = 2$.

Frequencies for Different Modes under CC Boundary Condition					
$\gamma$	$\chi$				
	0.2	0.3	0.5	0.8	0.9
**Mode 1**					
0.0	18.092	18.286	19.347	20.284	20.305
0.2	17.598	17.808	19.068	20.160	20.181
0.3	17.363	17.581	18.967	20.155	20.176
0.4	17.145	17.372	18.909	20.207	20.228
0.5	16.955	17.193	18.912	20.341	20.362
0.6	16.814	17.066	19.009	20.593	20.613
0.7	16.758	17.025	19.253	21.021	21.041
0.8	16.851	17.141	19.740	21.724	21.745
**Mode 2**					
0.0	48.617	48.800	49.000	49.315	49.353
0.2	46.217	46.420	46.664	47.030	47.067
0.3	44.888	45.102	45.376	45.773	45.811
0.4	43.448	43.673	43.983	44.420	44.458
0.5	41.866	42.104	42.460	42.947	42.984
0.6	40.098	40.349	40.764	41.316	41.353
0.7	38.066	38.329	38.825	39.469	39.505
0.8	35.619	35.890	36.504	37.290	37.327
**Mode 3**					
0.0	94.232	94.309	94.397	94.520	94.561
0.2	88.032	88.119	88.239	88.393	88.434
0.3	84.833	84.926	85.068	85.244	85.285
0.4	81.562	81.662	81.835	82.037	82.079
0.5	78.219	78.327	78.543	78.779	78.821
0.6	74.813	74.928	75.209	75.491	75.532
0.7	71.376	71.500	71.882	72.232	72.271
0.8	68.021	68.156	68.718	69.176	69.213

**Table 16:** Variation of the first three modal frequencies for a CS (2D-FGM) Beam with Changes in $\chi$ and $\gamma$, $n_x = 3$, $n_y = 4$, $\eta = 100$, and $n = 2$.

Frequencies for Different Modes under CS Boundary Condition					
$\gamma$	$\chi$				
	0.2	0.3	0.5	0.8	0.9
**Mode 1**					
0.0	11.442	11.599	12.681	14.366	14.505
0.2	10.657	10.826	12.104	14.100	14.253
0.3	10.235	10.410	11.809	13.999	14.161
0.4	9.7921	9.9733	11.510	13.929	14.102
0.5	9.3278	9.5150	11.210	13.901	14.086
0.6	8.8427	9.0350	10.905	13.927	14.128
0.7	8.3394	8.5343	10.589	14.020	14.241
0.8	7.8199	8.0115	10.224	14.183	14.432
**Mode 2**					
0.0	38.387	38.594	38.994	39.209	39.302
0.2	36.350	36.579	37.085	37.320	37.416
0.3	35.232	35.474	36.053	36.299	36.396
0.4	34.029	34.286	34.961	35.217	35.314
0.5	32.723	32.998	33.798	34.066	34.161
0.6	31.283	31.580	32.556	32.833	32.925
0.7	29.659	29.987	31.221	31.503	31.588
0.8	27.755	28.125	29.771	30.048	30.121
**Mode 3**					
0.0	80.310	80.417	80.495	80.636	80.690
0.2	74.835	74.958	75.063	75.247	75.303
0.3	72.028	72.159	72.284	72.498	72.555
0.4	69.171	69.311	69.463	69.717	69.775
0.5	66.270	66.419	66.607	66.915	66.975
0.6	63.339	63.495	63.734	64.121	64.182
0.7	60.415	60.578	60.895	61.402	61.464
0.8	57.617	57.779	58.227	58.940	59.004

**Table 17:** Variation of the First Three Modal Frequencies for a CF (2D-FGM) Beam with Changes in $\chi$ and $\gamma$, $n_x = 3$, $n_y = 4$, $\eta = 100$, and $n = 2$.

Frequencies for Different Modes under CF Boundary Condition					
$\gamma$	$\chi$				
	0.2	0.3	0.5	0.8	0.9
**Mode 1**					
0.0	2.1057	2.1610	2.7471	5.1833	6.3565
0.2	1.9964	2.0434	2.5629	4.9843	6.2422
0.3	1.9212	1.9630	2.4361	4.8401	6.1483
0.4	1.8259	1.8616	2.2773	4.6592	6.0242
0.5	1.7026	1.7315	2.0775	4.4376	5.8673
0.6	1.5395	1.5607	1.8249	4.1748	5.6784
0.7	1.3179	1.3311	1.5048	3.8778	5.4654
0.8	1.0088	1.0146	1.0998	3.5671	5.2464
**Mode 2**					
0.0	15.7970	15.9800	17.0030	17.8280	17.8470
0.2	14.2840	14.4820	15.7520	16.8930	16.9050
0.3	13.4980	13.7030	15.1340	16.4860	16.4970
0.4	12.6920	12.9060	14.5280	16.1410	16.1520
0.5	11.8700	12.0930	13.9430	15.8780	15.8910
0.6	11.0380	11.2710	13.3900	15.7250	15.7430
0.7	10.2130	10.4530	12.8770	15.7160	15.7430
0.8	9.4175	9.6605	12.3920	15.8880	15.9260
**Mode 3**					
0.0	46.7020	46.8960	47.1350	47.3640	47.3760
0.2	43.5960	43.8180	44.1320	44.3920	44.4050
0.3	41.9270	42.1640	42.5320	42.8090	42.8230
0.4	40.1540	40.4080	40.8460	41.1420	41.1560
0.5	38.2490	38.5220	39.0530	39.3690	39.3840
0.6	36.1640	36.4600	37.1230	37.4580	37.4740
0.7	33.8260	34.1500	35.0110	35.3620	35.3780
0.8	31.0860	31.4470	32.6430	32.9950	33.0100

## Conclusion

This study presents a robust discrete physical model (DPM) for investigating the small-amplitude transverse vibrations of non-uniform beams made from bidirectional functionally graded materials (2D-FGM), supported on elastic foundations. The model effectively captures the complexities associated with varying mechanical properties, geometric parameters, and boundary conditions. By representing the beam system as a multi-degree-of-freedom (MDOF) system, it provides a detailed and versatile framework for analysing vibrational behaviour.

The findings demonstrate that the natural frequencies and mode shapes are significantly influenced by the material gradation in both the *x* and *y* directions. In particular, increasing the gradation indices $n_x$ and $n_y$ results in a decrease in vibrational frequencies, due to the impact on mass and structural stiffness. This insight is crucial for tailoring material composition in applications that require specific vibrational characteristics.

Geometric taper, represented by the taper ratio $\gamma$, also plays a vital role in the vibrational behaviour. An increase in $\gamma$ leads to a linear reduction in frequencies, regardless of the boundary conditions (SS, CC, CS, CF), highlighting the critical influence of geometric non-uniformity. Moreover, the variation in frequencies across different boundary conditions offers valuable information for optimising design choices based on support configurations.

The study further emphasises the importance of boundary conditions in determining vibrational properties. Clamped-clamped (CC) beams exhibit the highest frequencies, reflecting greater stiffness, while clamped-free (CF) conditions lead to lower frequencies, indicating increased flexibility. The ranking of frequency values for different boundary conditions provides practical guidance for engineers in choosing appropriate configurations for specific applications.

Additionally, the elastic foundation’s stiffness, captured by the parameter $\eta$, has a noticeable impact on the vibrational frequencies, particularly in the lower modes. As $\eta$ increases, the first frequency rises significantly, indicating enhanced stiffness due to the foundation’s support. This effect becomes more pronounced with larger foundation lengths $\chi$. Furthermore, variable stiffness foundations $\alpha$ influence frequency trends, especially at higher conicity levels, underscoring the interaction between foundation properties and beam geometry.

The parametric studies conducted in this research, focusing on factors such as gradation indices, foundation stiffness, taper ratio, and boundary conditions, illustrate the flexibility and applicability of the model. In non-uniform beams with elastic foundations, the first vibrational frequency shows high sensitivity to the length and stiffness of the foundation. These findings enable targeted adjustments to the physical and geometric properties of beams, making the model a valuable tool for optimising performance in engineering applications, such as aerospace and civil structures.

Finally, the validity of the proposed DPM is confirmed through comparison with existing studies. The model accurately captures the dynamic behaviour of 2D-FGM beams under various boundary and support conditions, confirming its utility for future research and practical applications. Overall, this research provides an efficient method for performing parametric analyses and tailoring beam properties to enhance performance, reliability, and efficiency in dynamic environments.

## Data Availability

All data generated and analysed during this study are included in this published article.

## List of symbols

*L*: Length of the beam
*h*(*x*): Beam thickness at position *x*
*b*(*x*): Width of the beam at position *x*
*S*(*x*): Cross-sectional area at position *x*
*I*(*x*): Second moment of area at position *x*
*n*: Exponent in the thickness profile ($n=1$ linear, $n=2$ parabolic)
$I_0$: Second moment of area at $x=0$
*E*(*x*, *y*): Young’s modulus at (*x*, *y*)
$\rho (x,y)$: Mass density at (*x*, *y*)
$E_m$: Young’s modulus of metal
$E_c$: Young’s modulus of ceramic
$\rho _m$: Density of metal
$\rho _c$: Density of ceramic
$I_0(x)$: Equivalent line mass (mass per unit length) at position *x*
$n_x, n_y$: Material gradation indices in the axial (*x*) and thickness (*y*) directions
$\gamma$: Taper ratio of the non-uniform beam
*N*: Number of lumped masses in the discrete model
*l*: Distance between two consecutive masses ($l = L/(N+1)$)
$m_r$: Lumped mass at node *r*
$C_r$: Rotational spring stiffness at node *r*
$k_r^f$: Vertical foundation spring stiffness at node *r*
$\eta$: Dimensionless stiffness of the foundation
$\alpha$: Parameter of spatial variation of the foundation
$\chi$: Reduced length / position of the foundation
$m_{ij}$: Components of the mass tensor
$k_{ij}^l$: Components of the linear stiffness tensor (bending)
$k_{ij}^f$: Components of the stiffness tensor of the elastic foundation
$\delta _{ij}$: Kronecker delta symbol
$[M]$: Mass matrix of the DPM
$[K]$: Total stiffness matrix
$\omega _{\textrm{sys}}$: Circular Natural frequency
$\omega _{\textrm{sys}}^*$: Dimensionless frequency parameter
